# An Integrative Genetic Study of Rice Metabolism, Growth and Stochastic Variation Reveals Potential C/N Partitioning Loci

**DOI:** 10.1038/srep30143

**Published:** 2016-07-21

**Authors:** Baohua Li, Yuanyuan Zhang, Seyed Abolghasem Mohammadi, Dongxin Huai, Yongming Zhou, Daniel J. Kliebenstein

**Affiliations:** 1 Department of Plant Sciences, University of California, Davis, One Shield Avenue CA 95616, USA; 2 Key Laboratory of Biology and Genetic Improvement of Oil Crops, Ministry of Agriculture, Oil Crops Research Institute, Chinese Academy of Agricultural Sciences, Wuhan 430062, China; 3 National Key Laboratory of Crop Genetic Improvement, College of Plant Science and Technology, Huazhong Agricultural University, Wuhan 430070, China; 4 Department of Plant Breeding and Biotechnology, Faculty of Agriculture, University of Tabriz, Tabriz, Iran; 5 DynaMo Center of Excellence, Copenhagen Plant Science Centre, University of Copenhagen, Thorvaldsensvej 40, DK-1871 Frederiksberg C, Denmark

## Abstract

Studying the genetic basis of variation in plant metabolism has been greatly facilitated by genomic and metabolic profiling advances. In this study, we use metabolomics and growth measurements to map QTL in rice, a major staple crop. Previous rice metabolism studies have largely focused on identifying genes controlling major effect loci. To complement these studies, we conducted a replicated metabolomics analysis on a *japonica* (Lemont) by *indica* (Teqing) rice recombinant inbred line population and focused on the genetic variation for primary metabolism. Using independent replicated studies, we show that in contrast to other rice studies, the heritability of primary metabolism is similar to Arabidopsis. The vast majority of metabolic QTLs had small to moderate effects with significant polygenic epistasis. Two metabolomics QTL hotspots had opposing effects on carbon and nitrogen rich metabolites suggesting that they may influence carbon and nitrogen partitioning, with one locus co-localizing with *SUSIBA2* (*WRKY78*). Comparing QTLs for metabolomic and a variety of growth related traits identified few overlaps. Interestingly, the rice population displayed fewer loci controlling stochastic variation for metabolism than was found in Arabidopsis. Thus, it is possible that domestication has differentially impacted stochastic metabolite variation more than average metabolite variation.

Metabolism is a central process required for the uptake of energy and nutrients to ensure an organism’s survival, reproduction, and development. Plants have hugely diverse and complex metabolic pathways with metabolites being generally categorized as primary metabolites or secondary metabolites^1^. Primary metabolites, including sugars, amino acids and lipids, are metabolites that are required for the survival of an individual cell by providing the necessary energy and building blocks. In contrast, secondary metabolites, including flavonoids, terpenoids and glucosinolates, are required for the viability of the organism within an environment to provide resistance against the associated biotic and abiotic stresses. This environmental function leads to secondary metabolites often being lineage-specific and their role in biotic interactions means that these compounds often have pharmaceutical benefits^2^.

Because metabolism is a key intermediary in any physiological or developmental process, it is essential to develop a detailed picture of the genetic basis of variation in plant metabolism^3^,^4^,^5^,^6^. The combination of high-throughput quantification of metabolites with quantitative genetic approaches has become a key approach to study the genetic basis of plant metabolism^7^,^8^,^9^. There are two major quantitative genetics avenues to study and identify the causal genes underlying the complex traits including metabolism, either genome-wide association study (GWAS) or Quantitative Trait Locus (QTL) mapping in structured populations. These approaches have different strengths and weaknesses causing them to be highly complementary approaches for quantitative genetics. Using both of these approaches has led to substantial progress in elucidating the genetic architecture underlying primary and secondary metabolism in several model plant systems. Studies in the model dicot plant Arabidopsis have shown that most primary metabolites have a highly polygenic basis with numerous causal loci that typically have an allelic substitution effect size of less than 30%. In contrast, secondary metabolites have a blend of a few large effect loci with an underlying suite of small effect loci^10^,^11^. The metabolic loci cluster into sets of hotspots that affect broad swathes of primary metabolism and show pairwise and higher order epistasis both with other loci in the nucleus as well as with genetic variation in the organellar genomes^6^,^12^. Metabolite QTL (mQTL) mapping effects in tomato and maize showed a similar pattern of polygenic moderate to small effect additive loci for primary metabolism^13^,^14^,^15^,^16^.

Another key model system for the analysis of quantitative genetic variation in metabolism is rice, a major staple crop plants in agriculture, and a model system for the monocots. Domesticated rice has been proposed to comprise two major subspecies, *japonica* grown mainly in temperate east Asia and upland areas of south and southeast Asia and *indica* grown mainly in lowland throughout tropical Asia, along with additional minor subspecies^17^. Quantitative analysis of rice metabolomics in a collection of back-cross inbred populations, recombinant inbred populations and GWAS collections identified a similar pattern that primary metabolites had smaller effect loci than secondary metabolites^18^,^19^,^20^,^21^. In contrast to the other species, the rice metabolite quantitative genetics suggested that most metabolites had higher heritability and that there was little analysis of how much variance is due to epistasis^18^,^19^. Specific to rice, the GWAS analysis suggested that the *japonica* and *indica* species were highly divergent in their metabolic profiles^19^. It is however possible that these contrasts to other species may be caused by differences in experimental design and a stronger reliance on GWAS that has difficulty in handling epistatic or transgressive traits^10^,^12^. Thus, there is a need to complement these GWAS comparisons of *japonica* to *indica* with a metabolomics analysis of a recombinant inbred line population to provide finer details on how divergent metabolism may be between the *japonica* and *indica* subspecies. RIL populations while having less entering genetic variation provide a stronger capacity to investigate transgression and epistasis than does GWAS^22^,^23^,^24^. Additionally, a key unresolved question is how metabolomics QTLs in rice may link to physiological or developmental traits like growth.

To further investigate the metabolomic architecture of rice, we conducted an integrated genetic study of rice leaf metabolism, stochastic variation and development traits using a RIL derived from the Lemont (*japonica*) and Teqing (*indica*) parents. This identified a highly polygenic system whereby the vast majority of QTL had small to moderate effects and there was extensive transgressive segregation of loci from the *japonica* and *indica* parents. The heritability in a replicated design was similar to that found for other plants in contrast to previous reports. Twelve statistically significant mQTL hotspots were identified, with two of them controlling the partition of carbon and nitrogen partition in the rice primary metabolism. These hotspots displayed epistatic interactions at a level that was similar to that found in Arabidopsis and stochastic variation in metabolism was independent of the mean metabolite accumulation suggesting that the underlying architecture is similar between the two species. Finally, two out of twelve metabolomic hotspots were linked with altered variation in growth and development of the plant.

## Results

### The Rice Population, Metabolite Distribution and Detection

To map metabolomic QTLs within rice, we utilized a recombinant inbred population from a ‘Lemont’ x ‘Teqing’ (LT-RIL) rice cultivar cross^25^. Lemont (PI 475833) is a US tropical *japonica* rice cultivar, while Teqing (PI 536047) is an *indica* cultivar from China, and the LT-RILs have been widely used in rice QTL mapping researches, including the study of element concentration in grain^26^, grain yield^27^, developmental traits^28^, and disease resistances^29^. This population has not been previously studied for metabolomic variation and provides a novel genetic comparison with other rice metabolomics studies. The LT-RIL population in our study has 280 lines, with 175 restriction fragment length polymorphism (RFLP) markers spanned across all 12 chromosomes.

To measure metabolite variation, we grew the LT-RIL population together with the parental lines in two independent experiments during the fall of 2011 at the University of California Davis. Each line was planted within two random complete blocks within each experiment providing 4 independent replicates per line for metabolite analysis. Leaf samples were harvested 5 weeks after sowing and metabolites was measured using GC-TOF at the West Coast Metabolomics Center at UC Davis (http://metabolomics.ucdavis.edu/)^30^,^31^. This analysis detected 512 metabolites commonly found amongst the LT-RIL including 172 metabolites that could be specifically identified (Supplemental Table S1). The 172 known compounds are mainly from the primary metabolism as expected utilizing GC-TOF^10^,^32^,^33^.

### Genetic impact of LT-RIL and parental variation on metabolism

To assess the impact of genetic variation in the Lemont and Teqing parents and the LT-RIL on metabolite accumulation, we built linear models comparing the genetic and experimental variation. We first utilized a linear model to assess the metabolite variation in solely the Lemont and Teqing parents showing that only 16 of the 512 metabolites had statistical support for differential accumulation in Lemont versus Teqing. These 16 metabolites included 11 unknowns as well as valine, piceatannol, cerotinic acid, phosphate and acetophenone (P < 0.05) (Supplemental Table S2). In comparison, using the same linear model with the LT-RIL found 214 metabolites that had a significant genetic impact on their accumulation (P < 0.05). Thus, these *japonica* and *indica* parental lines have largely the same metabolomic profile for the detectable primary metabolism in our platform but the LT-RIL progeny expose extensive transgressive segregation underlying this similarity in the parental metabolomes.

**Figure 1:**
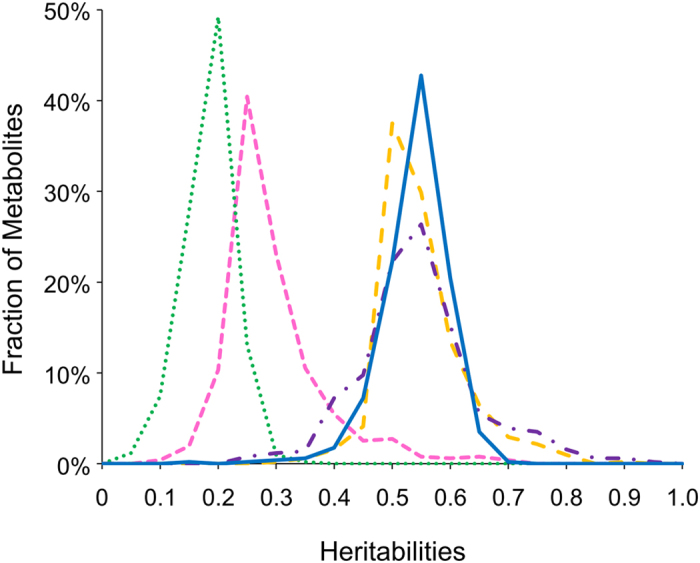
Metabolomic Heritabilities from the Rice Lemont and Teqing LT-RIL Population. Frequency plots of broad sense heritabilities of the metabolites are shown. The metabolite heritabilities are measured using the following datasets: metabolite accumulation in the combined experiments (pink square dot), metabolite accumulation in only experiment 1 (orange dash line), metabolite accumulation in only experiment 2 (purple dash dot), and heritability of -within line CV for all metabolites (Blue solid line). In addition, the variation due to genotype x experiment variation per metabolite is shown (green round dot).

In addition to the genetic variation, we utilized the linear model to estimate the effect of the uncontrolled differences between the two independent experiments upon the metabolite accumulation in the LT-RIL population. This showed that 422 metabolites had a significant influence of experimental variation on their accumulation but this did not greatly influence the genotype effect with only 42 of the 214 metabolites that had a genotype effect also having a genotype x experiment interaction effect (Supplemental Fig. S1 and Supplemental Table S2). Thus, the majority of these metabolites genetic variation is independent of the experiment. To compare heritability with other rice and plant metabolite QTL experiments, we estimated broad-sense heritability within each single experiment and across the combined experiments. Using the individual experiments, we found the median metabolite heritability of experiment 1 and experiment 2 are very similar, 0.507 and 0.513, respectively. This within experimental heritability of rice metabolites is similar to comparable previous rice studies of metabolomics variation in LT-RIL population that did not replicate across independent experiments but is significantly higher than the heritability found within Arabidopsis experiments that utilized multiple experiments^10^,^12^,^18^. To test if this difference is solely due to experimental variance, we estimated the broad-sense heritability using the combined experiments and this showed a significant decrease in broad-sense heritability, median of 0.248 (Fig. 1). This median heritability is similar to that found in Arabidopsis and other species indicating that the previously reported high heritability in rice metabolomics was simply a complication of not having replicated randomized experiments^10^,^12^,^18^.

### Metabolomic QTLs

**Figure 2:**
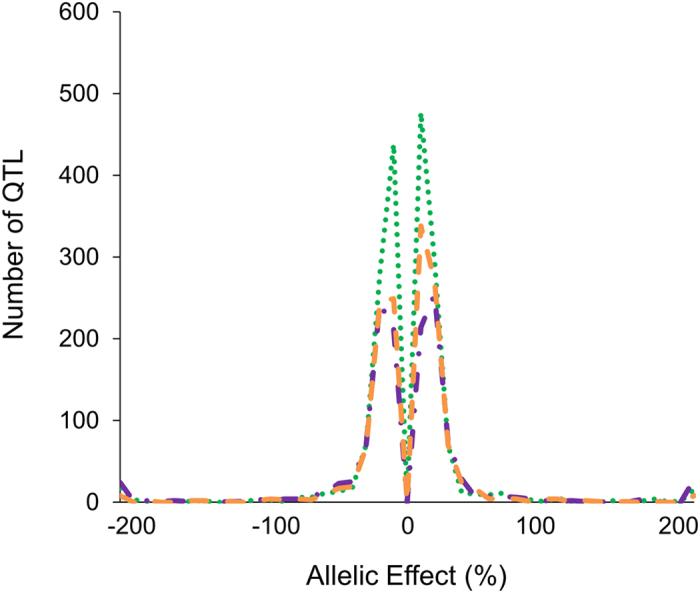
Distribution of Metabolite QTL Effect Sizes. The allelic effect for each locus for each metabolite was calculated by subtracting the average trait value for the lines with the Teqing genotype at a QTL from the average trait value for the lines with the Lemont genotype at the same QTL, then divided by the average trait value across all LT-RIL to standardize the allelic effect estimate. Effect sizes for QTLs found using the combined experiments are shown with a green round dot, metabolomics from only experiment 1 is shown with an orange dash line and metabolomics from only experiment 2 is shown with a purple dash dot.

The average accumulation of the 512 metabolites traits in the LT-RIL were utilized for the following QTL mapping effort. Because there was a significant effect of experimental variation between experiments on the genetics controlling metabolic accumulation, we conducted the QTL mapping with the means of each metabolites calculated from the combined experiments, experiment 1 and experiment 2 separately. This allows us to assess how the differences between the experiments may alter our ability to detect loci. QTL mapping for all metabolites was conducted using the R/qtl package with a two-step model involving a multi-QTL model^34^. This analysis identifed1797 QTLs for 202 metabolite traits using the data from the combined experiments, 1282 QTLs for 152 metabolite traits in experiment 1 and 1394 QTLs for 161 metabolite traits in experiment 2. In total, we detected QTLs for 285 of the 512 metabolites with an average of 8.5 QTLs per metabolite (Supplemental Table S3). The additive effects of the detected QTLs are generally small, with greater than 90% of all QTLs having less than a 50% additive change in metabolite accumulation (Fig. 2). There is a roughly symmetric distribution on the QTL effects caused by the *japonica* and *indica* alleles with a slight trend towards the Lemont (*japonica*) allele having a positive effect (Fig. 2). This lack of directional bias in additive effect of the *japonica* versus *indica* allele agrees with the previous observation of significant transgressive variation within this population. Thus, while there is genetic differentiation between the two parents, this is not caused by a general directionality in how the independent domestication histories have shaped the general metabolomes of these *japonica* and *indica* parents.

### Metabolic QTL Clusters

**Figure 3:**
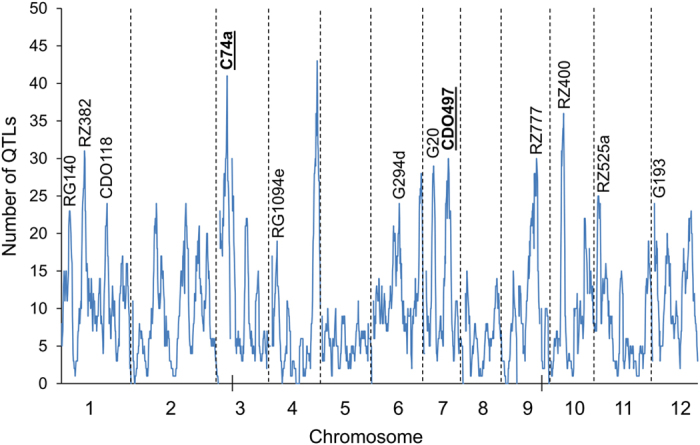
The Metabolite QTL Hotspots. The number of metabolite QTLs per chromosomal position is plotted using a 10 cM sliding window using the combined experiments. The closest marker names are provided for the twelve validated hotspots. The black lines on the x-axis for chromosomes 3 and 9 indicate gaps in the genetic maps for the LT-RIL population. The underlined markers C74a and CDO497 show the two hotspots potentially affecting carbon and nitrogen relationships.

To identify genomic hotspots controlling metabolomic variation in the LT-RIL, we plotted the QTL position of all metabolites across the rice genome using the QTLs identified in the combined experiment, experiment 1 and experiment 2 metabolite accumulations. This analysis identified 10, 11, and 10 hotspots from the combined experiment, experiment 1 and 2, respectively (Permutation significance thresholds using P ≤ 0.05 were 25, 19 and 20 QTL per position respectively) (Fig. 3 and Supplemental Figs 2–4). Combining the data from the three hot-spot surveys suggested 19 unique hotspots which we then proceeded to test against all metabolites using a linear model that utilized the marker closest to each hotspot peak as a factor in the model in combination with the experiment term. The inclusion of the experiment was used to help us assess the role of variation between the two experiments. This model with 19 hotspot markers and an experiment term was tested against all the metabolites and the data randomized and permuted to assess if a hotspot was indeed valid^10^,^12^. This analysis indicated that there was only strong support for 12 of the 19 hotspots (Fig. 3, Supplemental Tables S4 and S5).

To explicitly test if the 12 metabolomic QTL hotspots were conditional on differences between the two experiments or were detected across both experiments, we changed the linear model to include a marker x experiment term (Supplemental Table S6). This showed that 10 of the 12 QTL hotspots have most metabolites significantly altered by the main effect of genotype rather than the interaction of experiment x genotype. Across these 10 loci, there are on average 63 metabolites significantly altered by the main effect of genotype with only 32 metabolites being significantly altered by the genotype x experiment interaction. In contrast, the RG140 and G193 loci were more dependent on genotype x experiment interactions with more metabolites altered in a genotype x experiment interaction (53 and 70 metabolites respectively) than by solely genotype (49 and 68). This demonstrates that genotype is the main factor controlling metabolomic variation for most metabolite QTL hotspots in this set of experiments but that there is also a substantial influence of the genotype x experiment interaction that has not frequently been accounted for in rice metabolite QTL experiments.

**Figure 4:**
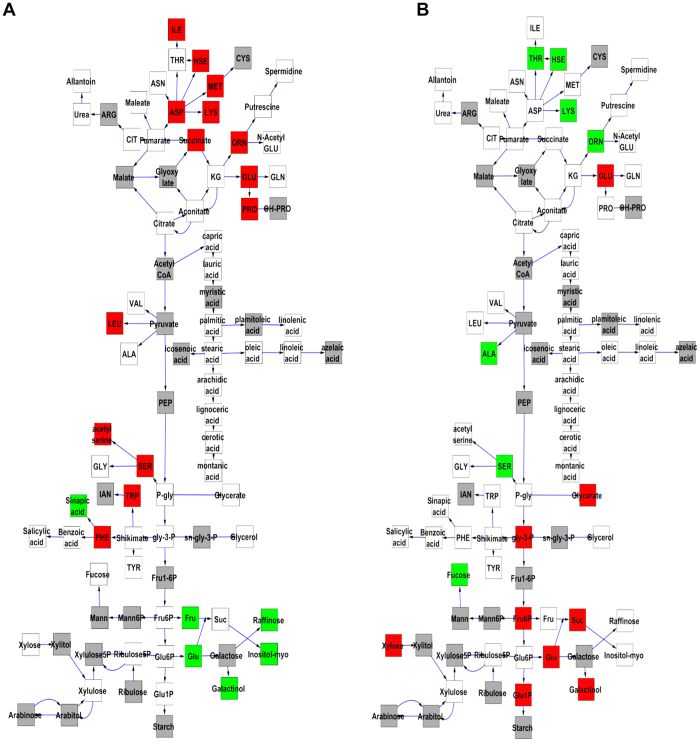
Metabolomic Consequence of Variation at Hotspots of C74A and CDO497. A map of central metabolism was created in Cytoscape and used to plot the estimated allele effect of genetic variation across primary metabolites. A red box shows increased metabolite accumulation when the line contains the Teqing allele while green shows increased metabolite accumulation when the line contains the Lemont allele of a QTL. White boxes are metabolites that were detected but not significantly influenced by the specific QTL and gray boxes show metabolites that were not detected. (**A**) Estimated allelic effects of the C74A hotspot across central metabolism. (**B**) Estimated allelic effects of the CDO497 hotspot across central metabolism.

We next proceeded to visualize how the different hotspots alter the primary metabolomic network. Using a network illustration of primary metabolism, we plotted the additive effects of an allelic substitution between Lemont and Teqing for all hotspots against primary metabolism (Fig. 4 and Supplemental Figs S1 and S5–S14). The most striking network effects on metabolism were displayed by the C74A hotspot on chromosome 3 and the CDO497 hotspot on chromosome 7. Both of these loci lead to an apparent rebalancing of major metabolites associated with carbon and nitrogen accumulation. For the C74A hotspot, the Teqing allele leads to increased accumulation of nitrogen-rich amino acids, including isoleucine, homoserine, methionine, aspartic acid, lysine, leucine, glutamic acid, serine and phenylalanine. In contrast, the Lemont allele leads to a corresponding decrease in the accumulation of diverse sugars including fructose, glucose, raffinose, myo-inositol and galactinol. The CDO497 hotspot showed an opposite pattern where the Teqing allele was low in amino acid accumulation but higher in sugars (Fig. 4 and Supplemental Table S6). The other hotspots had more dispersed effects on primary metabolism (Supplemental Figs S5–S14). This suggests that the C74A and CDO497 loci potentially influence the internal balance of carbon and nitrogen metabolites.

### Pairwise Epistasis for Metabolic QTL Hotspots

**Figure 5:**
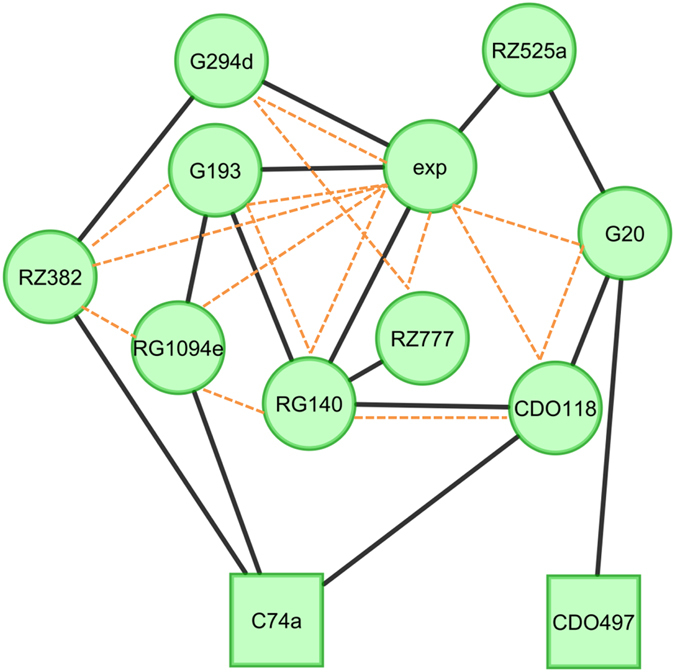
Epistatic Networks Influencing Rice Metabolic Variation. Solid lines show significant epistatic interactions between QTL hotspots are shown as edges linking the QTLs as nodes. QTL hotspots are named by the closest markers. Dashed lines show pairwise epistatic interaction between QTL hotspots is significantly influenced by experimental differences between the two experiments.

**Figure 6:**
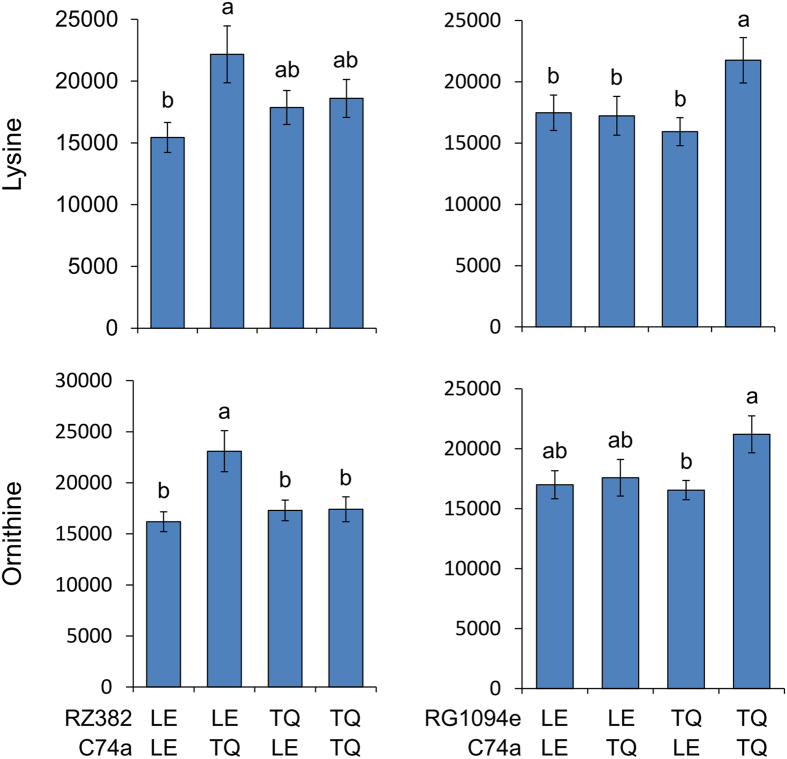
Epistasis Influencing Lysine and Ornithine. Boxplots showing the average ± SE accumulation of Lysine or Ornithing across tje four allelic classes for pairwise combinations of two QTL hotspots. Each allelic class is was represented by minimally 70 lines. Letters indicate statistically significant differences at P < 0.05 using Tukey’s post-hoc test.

Previous work in Arabidopsis has shown that epistatic interactions amongst metabolomic QTLs plays a major role in controlling primary metabolomic variation but this has been less apparent in rice studies predominantly focused on analyzing secondary metabolite accumulation^6^,^10^,^11^,^12^,^18^. Potential differences between the rice and Arabidopsis studies that could explain this discrepancy are the experimental design and the technical metabolomic platform. As our study utilized the same experimental design and technical platform as the previous Arabidopsis studies, we proceeded to test the level of epistasis that influences rice primary metabolism. To test the scale and the effect of epistatic interaction between the 12 metabolomic hotspots, we used linear models to test all potential interactions between hotspots and between the experiment and the epistatic terms for all metabolites (Supplemental Table S7). Using our previously published permutation assessment of which interactions occur more than expected by random chance^10^, 22 of the hotspot epistatic interactions were identified to control accumulation for more metabolites than expected randomly (Fig. 5 and Supplemental Table S4). Plotting the epistatic interactions showed that the loci were highly interconnected with a number of the epistatic interactions being significantly altered by a three-way interaction with experiment (Fig. 5 and Supplemental Table S7). There were no apparent central hubs in this epistatic network as nearly all of the loci interacted with 2–3 other loci suggesting that the genetic control of primary metabolism in rice is as epistatic as that previously found for Arabidopsis. The metabolites showed a wide range of epistatic patterns (Fig. 6, Supplemental Table S7). Interestingly, the two hotspot regions controlling the partitioning of carbon and nitrogen metabolites, C74a and CDO497 did not interact (Fig. 5). C74a interacted with RZ382, RG1094e and CDO118, while CDO497 interacted only with G20. This would suggest that the two underlying loci likely function to control carbon and nitrogen metabolite accumulation via separate genetic pathways.

**Figure 7:**
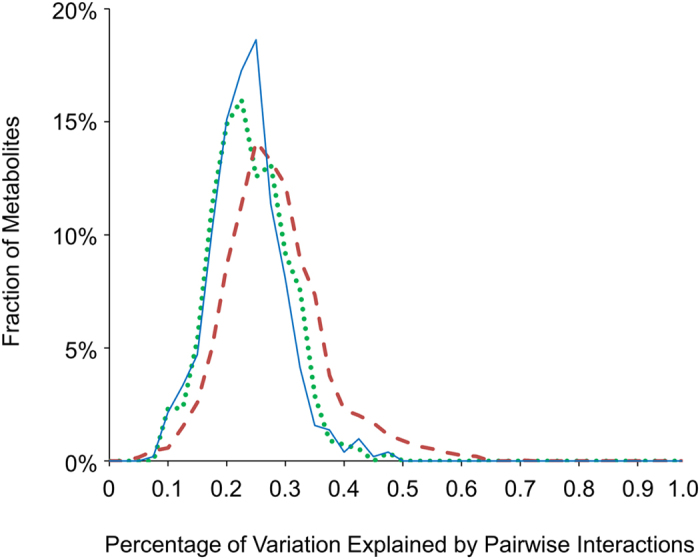
Comparison of Pairwise Epistasis between Arabidopsis and Rice Population. Frequency plots showing the percentage of variation explained by pairwise epistasis between metabolomic QTL hotspots from the rice Lemont-Teqing population (blue line), the Arabidopsis Kas-Tsu population (red dash line)^12^, and the Arabidopsis Bay-Sha Population (green round dot)^10^.

To directly compare the level of epistasis in the Arabidopsis and rice populations, we calculated the percentage of variation explained by the significant pairwise epistasis between the hotspots^10^,^12^. Interestingly, this showed that the scale of epistasis is similar and comparable in the rice and Arabidopsis populations for primary metabolism (Fig. 7). This suggests that the previously reported discrepancy between the level of epistasis in rice and Arabidopsis is either a result of experimental design or technical platform^18^. Taken together, the analysis showed the importance of the epistatic interactions in regulating the accumulation of the plant metabolism in both wild dicots and domesticated monocots. The one difference between the two species is that there was no significant three-way epistasis in rice in contrast to Arabidopsis. This could however be caused by differences in population size and remains to be assessed.

### QTLs for Metabolite Stochastic Variation

Stochastic phenotypic variation within a genotype is an important strategy for organisms to cope with the fluctuating experimental challenges^35^,^36^. Genetic variation of stochastic fluctuations in phenotypes has been shown to significantly influence the genetic basis of Arabidopsis metabolic variation^37^,^38^. To compare the rice metabolomics to Arabidopsis, we calculated the coefficient of variance (CV) for each metabolic trait as the measurement of genotypic stochastic variation^38^. CV is a dimensionless value and readily comparable across different model systems using a similar experimental design. Using the same QTL mapping procedure as described above, we were able to map 89, 69 and 53 QTLs from combined experiment, experiment 1 and experiment 2 that controlled CV of specific metabolites. This showed that we identified far fewer CV QTLs per metabolite than QTLs for average metabolite accumulation (Supplemental Table S3). As the heritability of the CV was similar to the heritability of metabolites traits (Fig. 1), the low identification rate of QTL for CV in the same rice population could be potentially due CV being more polygenic^39^.

**Figure 8:**
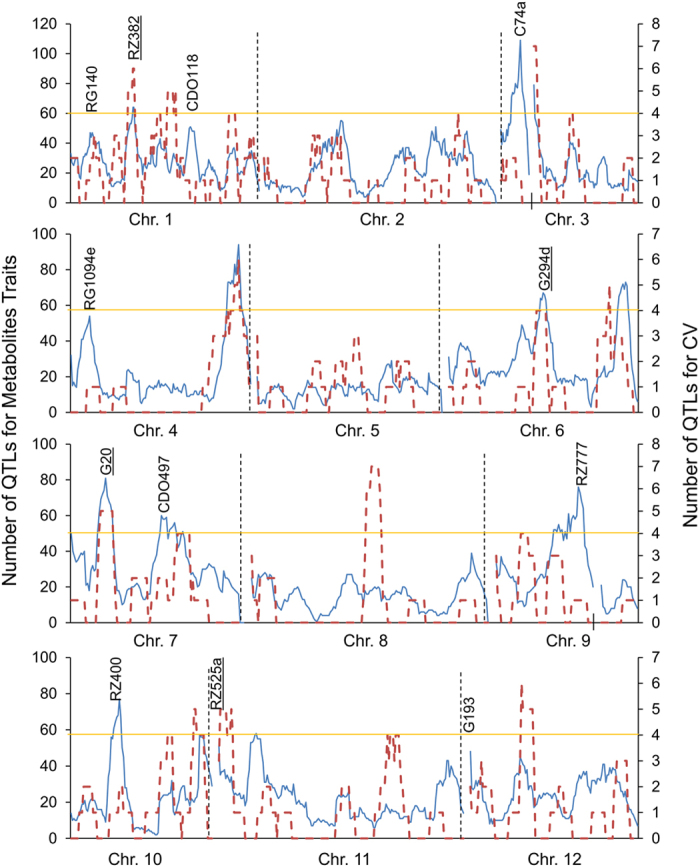
The Distribution of QTLs for Stochastic Metabolic Variation. The number of metabolite QTLs per chromosomal position is plotted using a a 10 cM sliding window using the combined experiments. The red dashed line shows QTL hotspots for Metabolite CV while the blue solid lines shows QTLs for average metabolite accumulation. QTL hotspots with the name underlined are those loci where there was overlap between hotspots for average and CV. The short black vertical lines on chromosomes 3 and 9 indicate gaps of the genetic maps on these two chromosomes. The horizontal lines show the permutation threshold for significant hotspots.

The rate of metabolic CV QTL identification in this rice population (average of 0.17 QTL per metabolite; 510 metabolites) is around one tenth the rate of CV QTL identification in a previous Arabidopsis study (average of 1.75 QTLs per metabolite; 414 metabolites). Both populations were of similar size and grown in a similar experimental design suggesting that there may be biological basis of the lower prevalence of CV QTLs in rice in comparison to Arabidopsis. Next we plotted all the QTLs of CV and metabolite traits from combined experiment, experiment 1 and experiment 2 together to compare hotspots found for CV and average metabolite accumulation (Fig. 8). By using the same permutation approaches described above, 19 hotspots were identified for the CV QTLs (threshold was 4, P < 0.05), and only 4 of them overlapped with the 12 metabolic hotspots. This showed that like in Arabidopsis, the genetic landscape of the average of the metabolites and their stochastic variation measured by CV was distinct (Fig. 8). Thus, CV of rice metabolic traits is a separate traits from the metabolic traits themselves, which is in accord with previous observation in Arabidopsis^38^.

### QTLs for Developmental Traits

**Figure 9:**
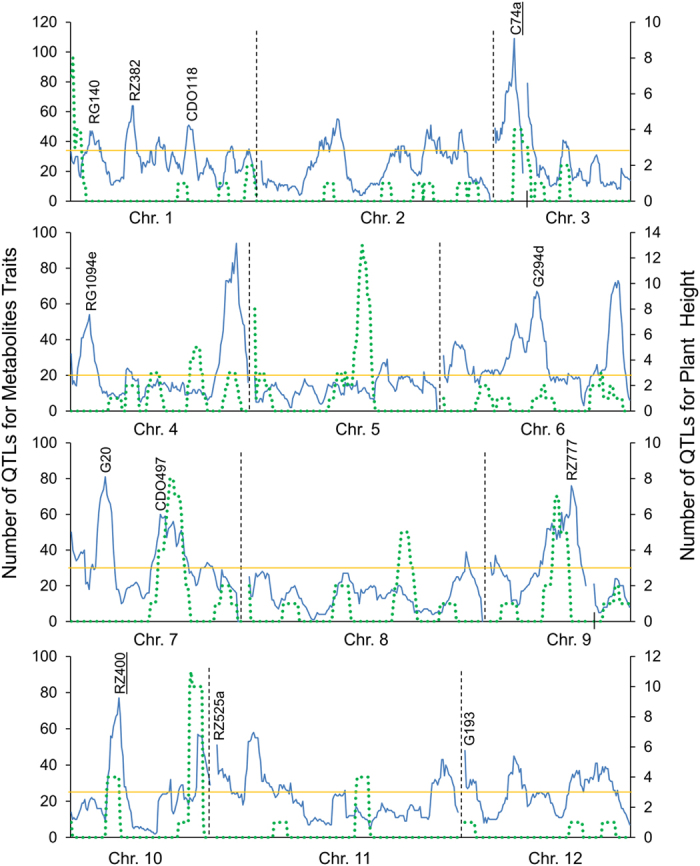
The Distribution of QTLs for Metabolites and Plant Height. The number of metabolite QTLs per chromosomal position is plotted using a 10 cM sliding window using the combined experiments. Metabolite QTL hotspots are shown with a solid blue line while those for plant height are shown using a line with green dots. QTL hotspots with the name underlined are those loci where there was overlap between hotspots for growth and metabolites. The short black vertical lines on chromosomes 3 and 9 indicated gaps of the genetic maps on these two chromosomes. The threshold of hotspot for plant height was 3 QTLs.

To compare the relationship between genetic variation for development and metabolomic QTLs across this LT-RIL population, we measured various developmental parameters in the same plants as used for the metabolomics. This included measuring plant height at different ontogenetic stages including measurements every two days from 11 days post germination to 37 days germination as well as the final matured plants. This provides 14 different temporally spaced measures of growth. For plant height, 32, 100 and 15 QTLs were identified from combined experiment, experiment 1 and experiment 2 respectively (Supplemental Table S3). This included a locus at the previously cloned *OsSPL14* locus for controlling rice plant height^40^. We also plotted all the QTLs of plant height and metabolite traits from combined experiment, experiment 1 and experiment 2 together for direct comparison of the loci influencing developmental and metabolic traits (Fig. 9). Using the permutation approaches described above, 16 hotspots were identified for plant height (threshold was 3, P ≤ 0.05), and only 2 of them overlapped with the metabolic hotspots. Estimating the additive effects of QTLs for plant height showed that there was transgressive segregation with both parents supplying alleles with positive impacts on growth (Supplemental Fig. S15). For tiller number, both at 5 weeks and 3 months, 5, 2, and 4 QTLs were identified from combined experiment, experiment 1 and experiment 2 respectively (Supplemental Table S3), including the previously confirmed and cloned loci on controlling tiller numbers, including *MOC1*^41^ and *TAD1*^42^. The overlap of our identified tillering QTLs with previously published cloned and confirmed loci suggests that our measurements are reproducible=.

To further explore the scale of the epistatic effect for the plant growth traits by the metabolite cluster markers, we used the same ANOVA model for metabolite traits to test the epistatic interactions of growth data. It showed that the variation explained by the epistatic interactions between the hotspots for the growth data (27.6 ± 1.1, Average ± SE of the fraction of total model variance explained by epistatic interactions) was statistically indistinguishable (p = 0.065, t-test) to the interactions of the hotspots of metabolite data (24.7 ± 0.3, Average ± SE) (Supplemental Fig. S16), indicating that epistatic interactions have comparable influence on both metabolism and plant growth in the rice population, albeit involving different suites of loci.

## Discussion

By studying a classic RIL population derived from *japonica* (Lemont) and *indica* (Teqing) in rice, we used quantitative genetics to work to integrate the genetic basis of phenotypic variation of rice metabolism, stochastic variation and development. The vast majority of primary metabolite QTLs had small to medium size effects that involved epistatic interactions similar to that found in other species. The use of a RIL population derived from a cross between the two major rice subspecies allowed us to show that there was extensive transgressive segregation for both metabolism and growth between these parents suggesting that it is possible to re-blend these two germplasms to create novel metabolic phenotypes. This transgressive segregation included several loci that may potentially affect the balance of carbon and nitrogen rich metabolites. We were also able to identify loci that specifically affected stochastic variation in metabolic phenotypes suggesting that it is possible to breed for metabolic stability separately from the average metabolite accumulation. The co-localization of the loci found in our study, both in primary metabolism and developmental traits, with the cloned and validated genes reinforced the accuracy and value of the findings in this study. Our findings also showed that discrepancies in heritability and epistasis with regards to previous rice research and other plant systems likely arise from differences in experimental design.

It was generally believed that genetic variation and diversities of most crop plants, including rice decreased during the domestication process because only a few of individuals from a wild progenitor species were used for the human selection process causing a bottleneck^43^. Domestication has made clears marks on the appearance of the domesticated plants with larger grains, more robust plants structure, increased apical dominance and a loss of natural seed dispersal, also known as “domestication syndrome”^44^. It remains to be explored if the genetic architecture of metabolism in domesticated crops is dramatically different than that in wild species. Interestingly, the genetic architecture of this rice population is highly similar to that found in wild species, Arabidopsis, and other crops, tomato and maize. Specifically, the heritability’s are similar, most QTLs are small to moderate effect with large effect loci being predominantly secondary metabolites and effect distributions are symmetric. Finally, there is evidence of epistasis in primary metabolism. Thus, there is no direct evidence of a domestication syndrome for metabolism within the existing data. This raises the possibility that while domestication has significantly altered development in domesticated crop plants, this has not dramatically altered the architecture of genetic variation controlling the average primary metabolite. The one trait that did show evidence of lower variation in the domesticated crop was the stochastic variation of metabolite accumulation. It remains to be tested how much these results are driven by differences in the evolutionary lineages of the rice and Arabidopsis progenitor’s versus how much is truly representing a difference in how domestication influences morphological versus metabolic traits.

Although the population structure of rice is clearly defined^17^,^45^ with *japonica* and *indica* as two major subspecies, the effect of this on genetic variation in plant metabolism in the two subspecies is just beginning to be assessed via recent QTL and GWAS studies^18^,^19^,^20^,^21^. The study of rice secondary metabolism using GWAS studies showed that there was extensive partitioning amongst the subspecies involving large effect loci^19^. This study did not specifically assess if primary metabolism alone showed the extensive subspecies partitioning. The Lemont and Teqing parents showed very few metabolites that were differentially accumulating while their progeny in the LT-RIL population showed that this was caused by extensive transgressive in primary metabolism. Further, the effects of the loci were largely symmetric. Thus, these *japonica* and *indica* parents do not support an extensive effect of the subspecies partitioning on primary metabolism. This raises the possibility of creating new primary metabolic phenotypes by introgressing loci between the two subspecies, potentially the two loci that may be linked to controlling the partitioning of Carbon and Nitrogen such as the locus that overlaps *SUSIBA2*/*WRKY78*^46^,^47^,^48^,^49^. If these results are generally indicative of the *japonica* and *indica* primary metabolism, the difference between primary and secondary metabolism could provide hints as to what was selected upon during domestication.

Rice is one of the most important staple crop plants in the world thus serving as one of the major source in providing human nutrition, including carbon and nitrogen. The optimal allocation of carbon and nitrogen in rice metabolism would potentially contribute to the high quality and production of rice, benefit billions of people, especially in developing countries, and is generally believed be controlled by multiple genes^50^. What’s more, the climate changes would have complex impacts on the carbon and nitrogen status with higher temperature and CO_2_ concentration in the coming decades^51^. So understanding the genetic control of the carbon/nitrogen partition in rice would help to address some of the most challenging problems in modern agriculture. In our study, we identified two hotspot regions, C74A hotspot on chromosome 3 and the CDO497 hotspot on chromosome 7, with opposite effects on carbon (sugar) and nitrogen (amino acid) containing metabolites that may influence the broader Carbon/Nitrogen partitioning (Fig. 4). Interestingly, the CDO497 locus co-localizes with *SUSIBA2* (Os07g0583700), a WRKY transcription factor (*WRKY78*), that regulates the carbon reallocation from amylose to amylopectin and proved to be a promising breeding locus for optimal carbon partition^46^,^47^,^48^,^49^. The dramatic effects of transgenic *SUSIBA2* on regulating rice primary metabolism and sink-source suggesting that this is a prime candidate for this locus^47^. In contrast, there were no known candidate genes underlying the C74A hotspot on chromosome 3 from our knowledge. Thus, the choice of the RIL population derived from *japonica* and *indica* gave us more power in detecting the metabolic QTLs underlying the genetic variation between the two subspecies, and the co-location of one of the hotspots with *SUSIBA2* highlights the potential value of the novel locus around hotspot C74, which would be worth to be further explored in the future study.

A key aim of quantitative and system biology is to bridge genetic connections between phenotypes^52^. Using our data, we were investigating if there are QTLs that co-localize for rice metabolism and growth. The landscapes of the mQTL and plant height QTL were distinct, and there were only two hotspots that showed co-localization between plant height QTLs and mQTLs (Fig. 9). This is similar to what was found when comparing QTLs for plant growth and metabolism in Arabidopsis. This could be interpreted as saying that the QTLs for growth and metabolism are different. However, further analysis of the Arabidopsis data showed that there was a connection between the traits that was not identifiable in the mapped QTLs. This was most likely caused by small RIL populations (<500) having a previously unrecognized false-negative error rate in QTL detection for polygenic traits^6^,^53^. To fully resolve the overlap in growth and metabolomic QTLs will require the development of vastly larger QTL or GWAS populations than are currently available.

## Summary

The large number of metabolite and growth QTLs found in this population and their transgressive nature suggests that there is significant potential for manipulating the rice metabolome by combining the *japonica* and *indica* lineages in future efforts. Critically the analysis of the metabolomic network effects of these loci showed that it may be potential to identify loci of interest for carbon/nitrogen partitioning. This also showed how investigating the metabolic network effects of loci may help to identify broader physiological consequences of these loci. It will be important to assess the ability of these loci to control Carbon/Nitrogen partitioning at both the whole plant and field level to test if they can be utilized in agronomic settings. Expanding these results will require the development of additional populations that are larger and include reciprocal crosses.

## Methods

### Growth of the Lemont × Teqing Rice RIL population

Seeds of the 280 lines of the Lemont × Teqing Rice recombinant inbred population together with parent lines were obtained^25^. The LT-RILs and the parental genotypes were grown in two separate experiments in the fall of 2011 within a greenhouse at the University of California, Davis. Within each experiment, two plants per LT-RIL line and two plants per each parental genotype were grown with one plant per each of two randomized complete blocks. This provides four independent replicates for most of LT-RIL lines, and each of the parental genotypes. Each independent plant was grown in a 10 cm (length) × 10 cm (width) × 9 cm (height) pot filled with UC Soil Mix (1 peat moss: 1 coarse sand (v:v)). Prior to sowing, the seeds were imbibed in water for the 3 days. We placed approximately 3–5 seeds from each line in the center of a pot and thinned the plants to leave one seedling per pot one week after sowing. The plants were watered twice a week with nutrient water prepared by UC Davis Research Greenhouses throughout the following study. The fertilizer supply was based on a packaged pre-mixed material from GrowMore, Inc with continuous feed into the irrigation supply allowing plants to receive a small amount of fertilizer at every irrigation. The macronutrient ratio for nitrogen:phosphorus:potassium was 2:1:2. No boron was added to the irrigation water as there is sufficient boron in the potting soil and water in the facility (http://greenhouse.ucdavis.edu/research/materials/mediafert.html).

### Metabolomics analysis

5 weeks after sowing, we harvested leaf material from each plant for metabolomics analysis. Briefly, for each of the randomized complete blocks, we harvested all of the samples within 2.5 hours surrounding the middle of the day. In a preliminary experiment with both parental genotypes and a random sampling of 10 RILs, we measured the weight of each leaf disk from each genotype in eight fold replication. ANOVA analysis of this showed no statistical evidence for genetic variation in the weight per leaf disk across the genotypes suggesting that this is an acceptable approach to speed up the harvest time and decrease any temporal bias while introducing minimal bias from density variation (P = NS). Three leaf discs from the middle of the most extended leaf blade including the midvein of each plant were taken, placed into 2 mL centrifuge tubes, snap frozen in liquid nitrogen, and stored at −70 before extraction. Metabolites were extracted as previously described data^10^,^12^. All metabolomic samples were run at the UC Davis Genome Center Metabolomics Facility using a GC-TOF per previously published protocols^30^,^31^. Metabolite identity was determined by comparing retention time and mass to the 2007 UC Davis Genome Center Metabolomics Facility metabolites database (http://fiehnlab.ucdavis.edu/Metabolite-Library-2007)^33^. For the control sample, a bulk sample was made by mixing aliquots from all the samples into a single sample, and this control was reinjected every 20 samples as per the UC Davis Genome Center Metabolomics Facility protocols^30^. The control sample was then then used to adjust the GC-MS response signals to account for any drift in the sensitivity of the MS and thus minimize any influence on the data caused by technical variation^30^,^31^. The adjusted ion count values were used as a surrogate for metabolite abundance. Metabolite abundance was median normalized prior to analysis to account for any technical variation between samples. In total, replicated metabolomics data were available for 238 lines of the rice LT-RIL population together with parent lines. The metabolites that were robustly called in all samples were further investigated for the QTL mapping effort in our study.

### Growth Phenotyping

After sowing, plant height was measured every 2 days for all individuals beginning from Day 11 until metabolomics harvest, Day 37. Post-harvest, we allowed the population to fully mature in the greenhouse and plant height was measured at the end of the growth. We also counted tiller numbers in each individual on day 37 (Tiller1), and at the end of growth (Tiller2). Means of the plant height and tiller numbers were used for QTL mapping.

### Estimation of Heritability

All the LT-RIL lines were represented in every block in both experiments creating a balanced randomized complete block design. Thus, we used a linear model to estimate the broad-sense heritability (H^2^) for all traits as 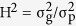, where 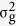 was the traits genetic variance from the LT-RILs and 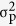 was the total phenotypic variance for a trait^8^. The ANOVA model (Linear heritability model) for each trait is shown in the Supplemental Table S3. In addition to the absolute metabolite accumulation, we also utilized the independent experiments to estimate the per-line CV for each metabolite as previously described^38^.

### QTL Analysis

QTL analysis was performed using the R/qtl package^34^ and the R software suite^54^. We applied a two-step methodology to identify significant QTLs. QTLs were initially identified by simple interval mapping using the Haley-Knott (hk) algorithm. Genome wide LOD significance thresholds were calculated by permutation test (1000 repetitions) for all QTL mapping steps and approaches. The identified QTLs were further by fitting a multi-QTL model via multiple interval mapping and dropping one QTL at a time using the scantwo function. Confidence intervals were calculated as 1.5- LOD support intervals. In addition, QTL and QTL x QTL interactions were assessed by building stepwise models for multiple QTL using forward selection and backward elimination to independently identify the best QTL model for each metabolite and growth trait. For all the QTL, percentage of variance explained, additive effect, standard error of additive effect, lower and higher confidence interval markers and lower and higher confidence interval genomic position were estimated within R/QTL.

### Additive ANOVA model

To directly test the additive effect of each identified QTL hotspots, we used an ANOVA model containing the markers most closely associated with each of the significant QTL hotspots as individual main effect terms together with experiment term. For each metabolite, the average accumulation in lines of genotype g at marker m was shown as y_gm_. The model (Additive Model) for each metabolite in each line (y_gm_) was: 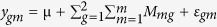, where *g* = Lemont (1) or Teqing (2); and the main effect of the markers was denoted as *M* involving 12 markers *m* = 1, …, m. The experiment term was included as an additional factor to test for experimental effects. We tested all metabolites with the appropriate model implemented in the R/car package, which returned all P values, Type III sums-of-squares for the complete model and each main effect, and QTL main-effect estimates (in terms of allelic substitution values)^54^,^55^.

### QTL epistasis analysis

To test directly for epistatic interactions between the detected QTL hotspots, we conducted an ANOVA using the pairwise epistasis model. Within the model, we tested all possible pairwise interactions between the markers near the hotspots. For each phenotype, the average value in the LT-RIL of genotype g at marker m was shown as y_gm_. The model for each metabolite in each line (y_gm_) was:


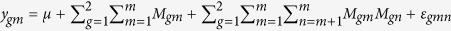


where *g* = Lemont (1) or Teqing (2); The main effect of the markers was denoted as *M* having a model involving 12 markers *m* = 1, …, m. and the identity and count of the second marker is represented by the *M*_*n*_ term. The experiment term was included as an additional factor to test for interactions between the experiment and hotspots. P values, Type III sums-of-squares for the complete model and each individual term and QTL pairwise-effect estimates in terms of allelic substitution values were obtained as described for marker model ANOVA^54^,^55^ Significance values were corrected for multiple testing within a model using FDR (<0.05) in the automated script. The main effect and epistatic interactions of the loci in each phenotypic class were visualized using Cytoscape.v2.8.3^12^,^56^.

## Additional Information

**How to cite this article**: Li, B. *et al*. An Integrative Genetic Study of Rice Metabolism, Growth and Stochastic Variation Reveals Potential C/N Partitioning Loci. *Sci. Rep.* **6**, 30143; doi: 10.1038/srep30143 (2016).

## Supplementary Material

Supplementary Figures S1-S16

Supplementary Table S1

Supplementary Table S2

Supplementary Table S3

Supplementary Table S4

Supplementary Table S5

Supplementary Table S6

Supplementary Table S7
