# Supplementary material for: An Integrative Genetic Study of Rice Metabolism, Growth and Stochastic Variation Reveals Potential C/N Partitioning Loci
Source: Sci Rep. 2016 Jul 21;6:30143. doi: 10.1038/srep30143 (PMC4954952; doi:10.1038/srep30143)
Supplement: Supplementary Figures S1-S16 [file srep30143-s1.pdf]

An Integrative Genetic Study of Rice Metabolism, Growth and Stochastic Variation Reveals Potential C/N Partitioning Loci

Baohua Li[1,+], Yuanyuan Zhang[1,2,3,+], Seyed Abolghasem Mohammadi[4,+], Dongxin Huai[2,3], Yongming Zhou[3], and Daniel J Kliebenstein[1,5,*]

[1]Department of Plant Sciences, University of California, Davis, One Shield Avenue CA 95616, USA

[2]Key Laboratory of Biology and Genetic Improvement of Oil Crops, Ministry of Agriculture, Oil Crops Research Institute, Chinese Academy of Agricultural Sciences, Wuhan 430062, China.

[3]National Key Laboratory of Crop Genetic Improvement, College of Plant Science and Technology, Huazhong Agricultural University, Wuhan 430070, China

[4]Department of Plant Breeding and Biotechnology, Faculty of Agriculture, University of Tabriz, Tabriz, Iran

[5]DynaMo Center of Excellence, Copenhagen Plant Science Centre, University of Copenhagen, Thorvaldsensvej 40, DK-1871 Frederiksberg C, Denmark

[+]These authors contributed equally to this work.

[*]Corresponding author

Emails: kliebenstein@ucdavis.edu (DJK)

Additional information

Competing financial interests

The authors have no competing interests as defined by Nature Publishing Group, or other interests that might be perceived to influence the results and/or discussion reported in this paper.

Supplementary Information (Figures)

Supplemental Figure S1. Influence of Experimental Variation on the Metabolome.

Supplemental Figure S2. Metabolite QTL Hotspots from Combined Experiments.

Supplemental Figure S3. The Metabolite QTL Hotspots from Experiment 1.

Supplemental Figure S4. The Metabolite QTL Hotspots from Experiment 2.

Supplemental Figure S5. Metabolomic Consequence of Variation at Hotspot RG140.

Supplemental Figure S6. Metabolomic Consequence of Variation at Hotspot RZ382.

Supplemental Figure S7. Metabolomic Consequence of Variation at Hotspot CDO118.

Supplemental Figure S8. Metabolomic Consequence of Variation at Hotspot RG1094e.

Supplemental Figure S9. Metabolomic Consequence of Variation at Hotspot G294d.

Supplemental Figure S10. Metabolomic Consequence of Variation at Hotspot G20.

Supplemental Figure S11. Metabolomic Consequence of Variation at Hotspot RZ777.

Supplemental Figure S12. Metabolomic Consequence of Variation at Hotspot RZ400.

Supplemental Figure S13. Metabolomic Consequence of Variation at Hotspot RZ525a.

Supplemental Figure S14. Metabolomic Consequence of Variation at Hotspot G193.

Supplemental Figure S15. Heat Map Showing the Location and Effect of Plant Height QTLs.

Supplemental Figure S16. Pairwise ANOVA Analysis with Plant Height and Tiller Numbers

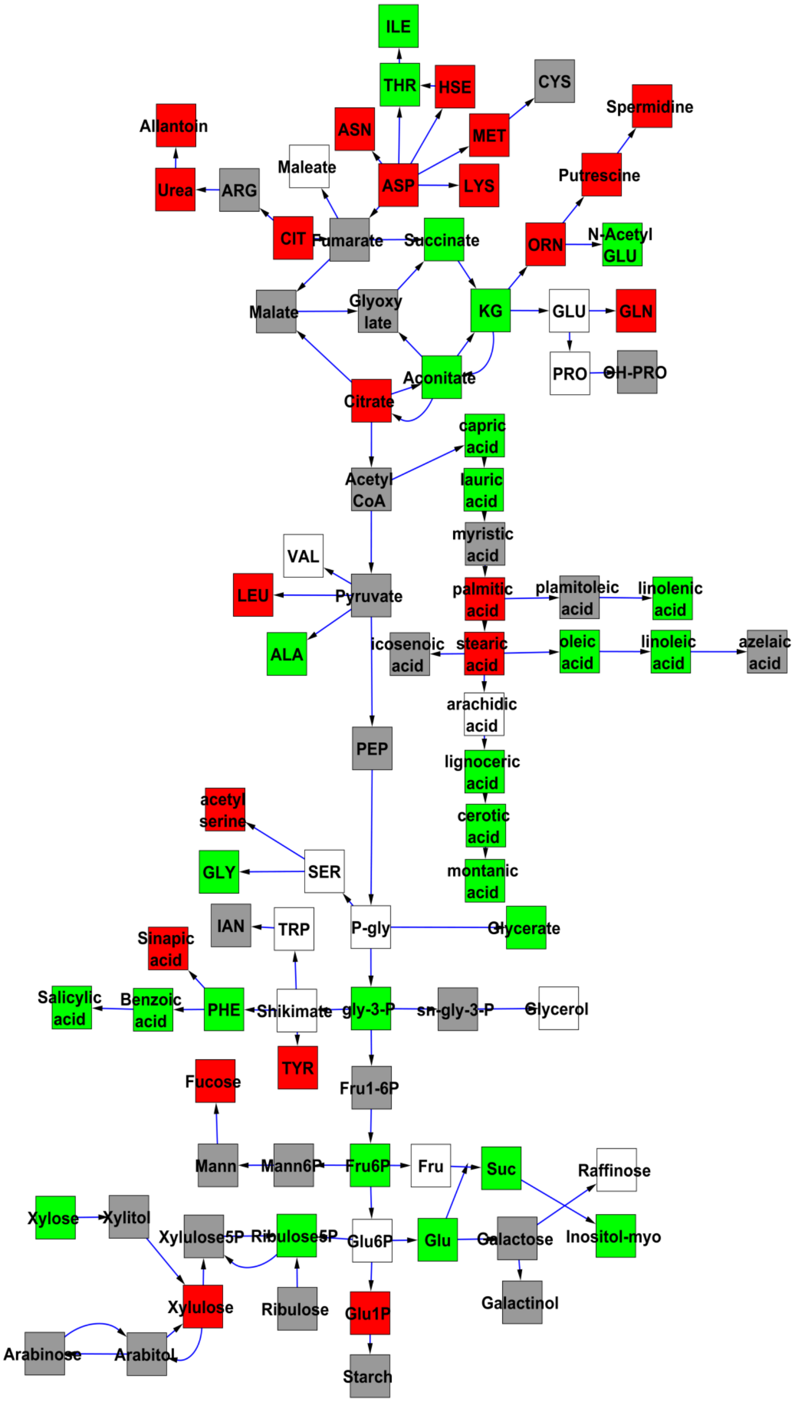

Supplemental Figure S1. Influence of Experimental Variation on the Metabolome.

A map of central metabolism was created in Cytoscape and used to plot the estimated effect of variation across the two experiments on primary metabolites. A red box shows increased metabolite accumulation when the line was grown in experiment 2 while green shows increased metabolite accumulation when the line was grown in experiment 1. White boxes are metabolites that were detected but not significantly influenced by the specific QTL and gray boxes show metabolites that were not detected.

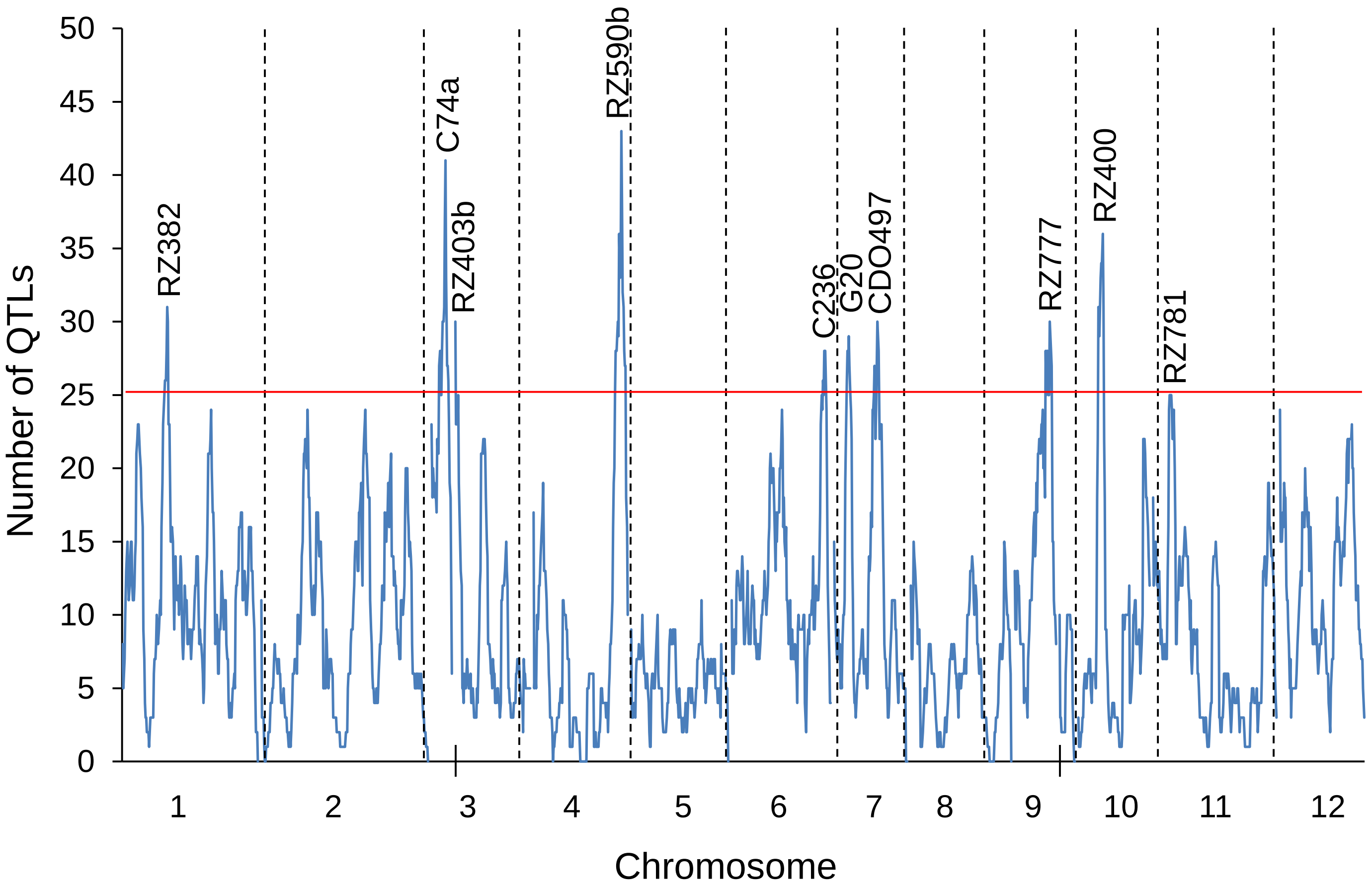

Supplemental Figure S2. Metabolite QTL Hotspots from Combined Experiments.

The number of metabolite QTLs per chromosomal position is plotted using a 10 cM sliding window using the combined experiments. Hotspots are labeled above the respective loci with the name of the marker closest to the peak. The red line shows the permutation significance threshold ($\alpha = 0.05$), and the black lines on the x-axis of chromosomes 3 and 9 show gaps in the genetic maps.

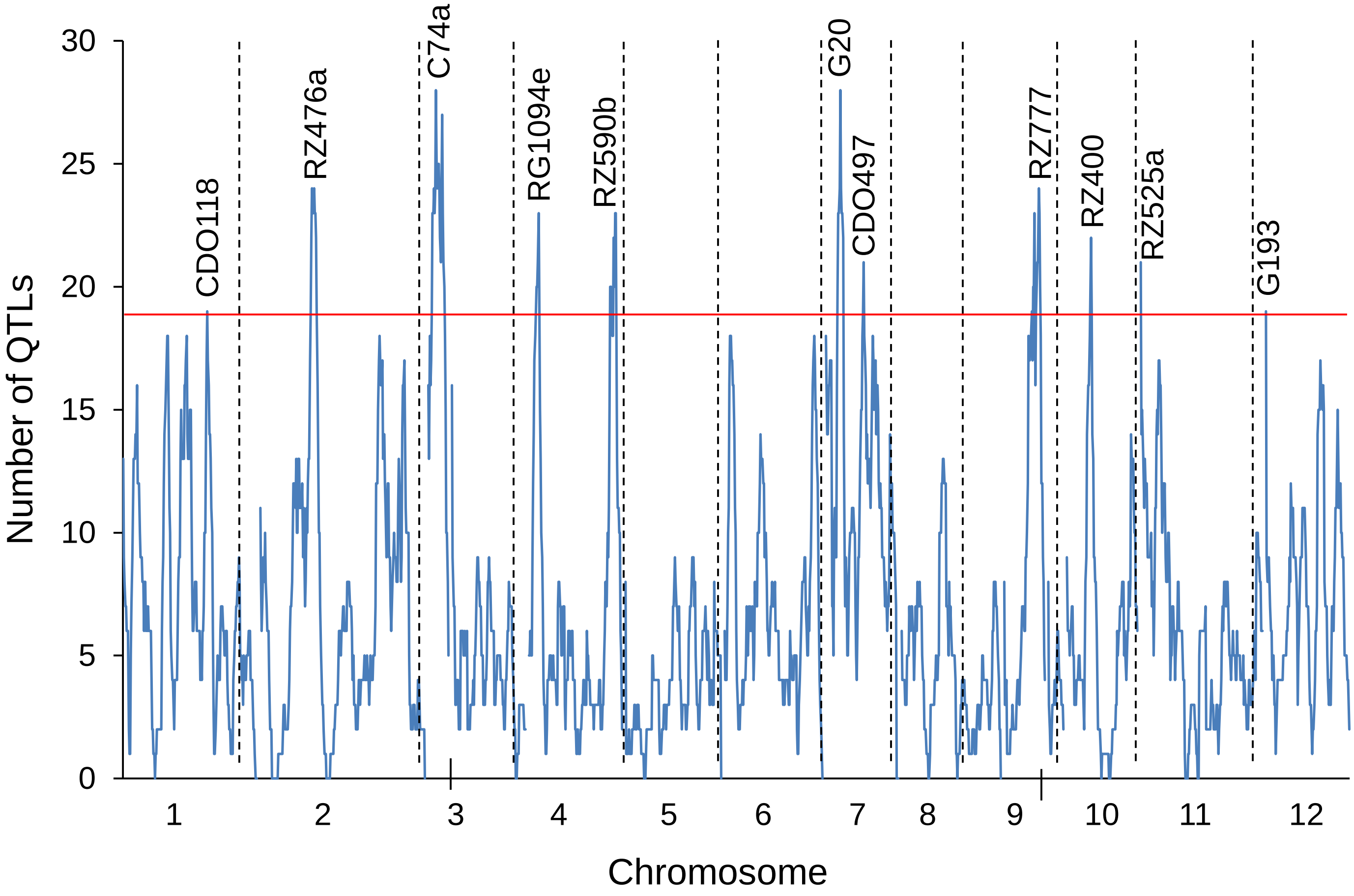

Supplemental Figure S3. The Metabolite QTL Hotspots from Experiment 1.

The number of metabolite QTLs per chromosomal position is plotted using a 10 cM sliding window using solely experiment 1. Hotspots are labeled above the respective loci with the name of the marker closest to the peak. The red line shows the permutation significance threshold ($\alpha = 0.05$), and the black lines on the x-axis of chromosomes 3 and 9 show gaps in the genetic maps.

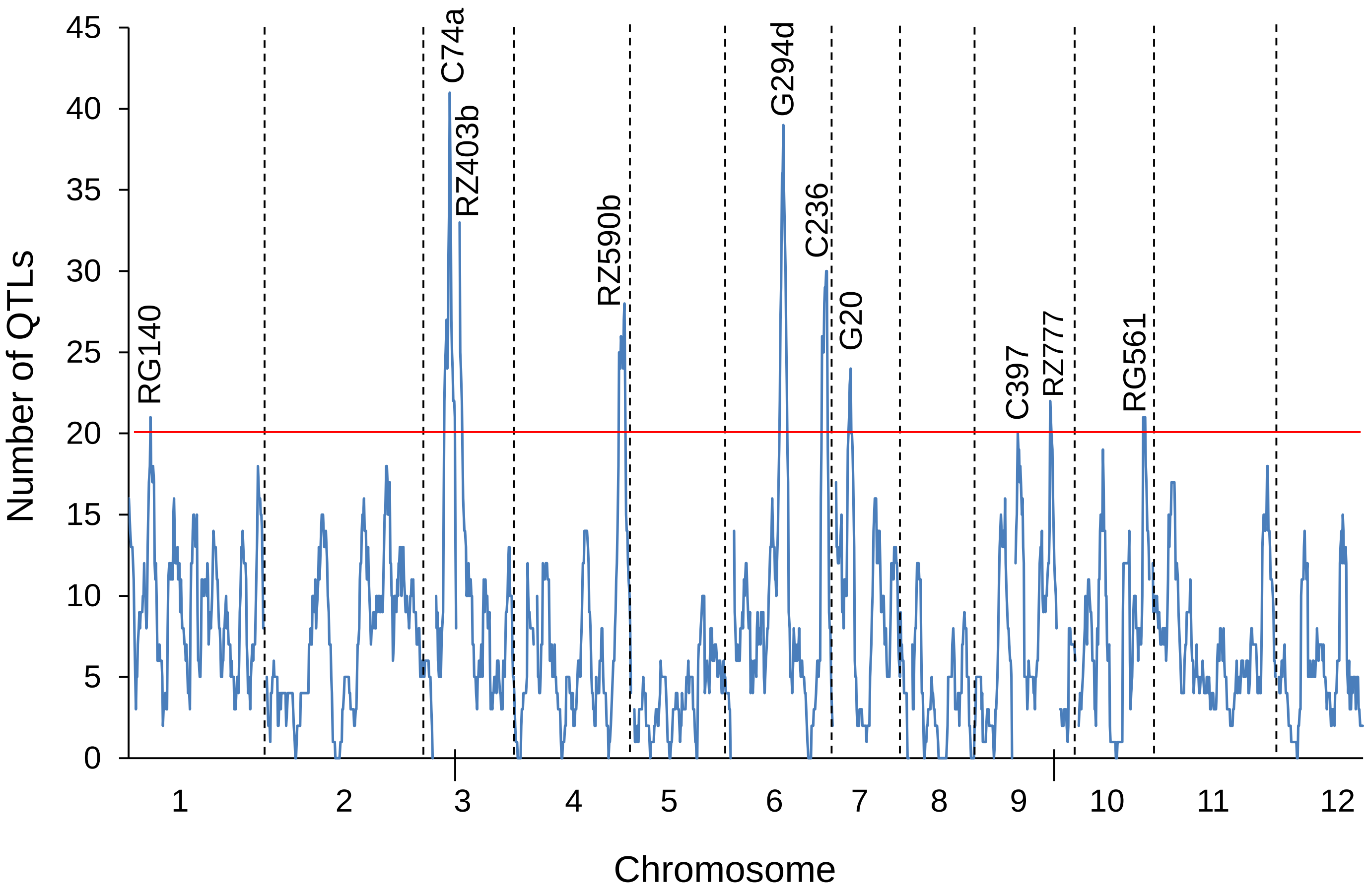

Supplemental Figure S4. The Metabolite QTL Hotspots from Experiment 2.

The number of metabolite QTLs per chromosomal position is plotted using a 10 cM sliding window using the using solely experiment 2. Hotspots are labeled above the respective loci with the name of the marker closest to the peak. The red line shows the permutation significance threshold ($\alpha = 0.05$), and the black lines on the x-axis of chromosomes 3 and 9 show gaps in the genetic maps.

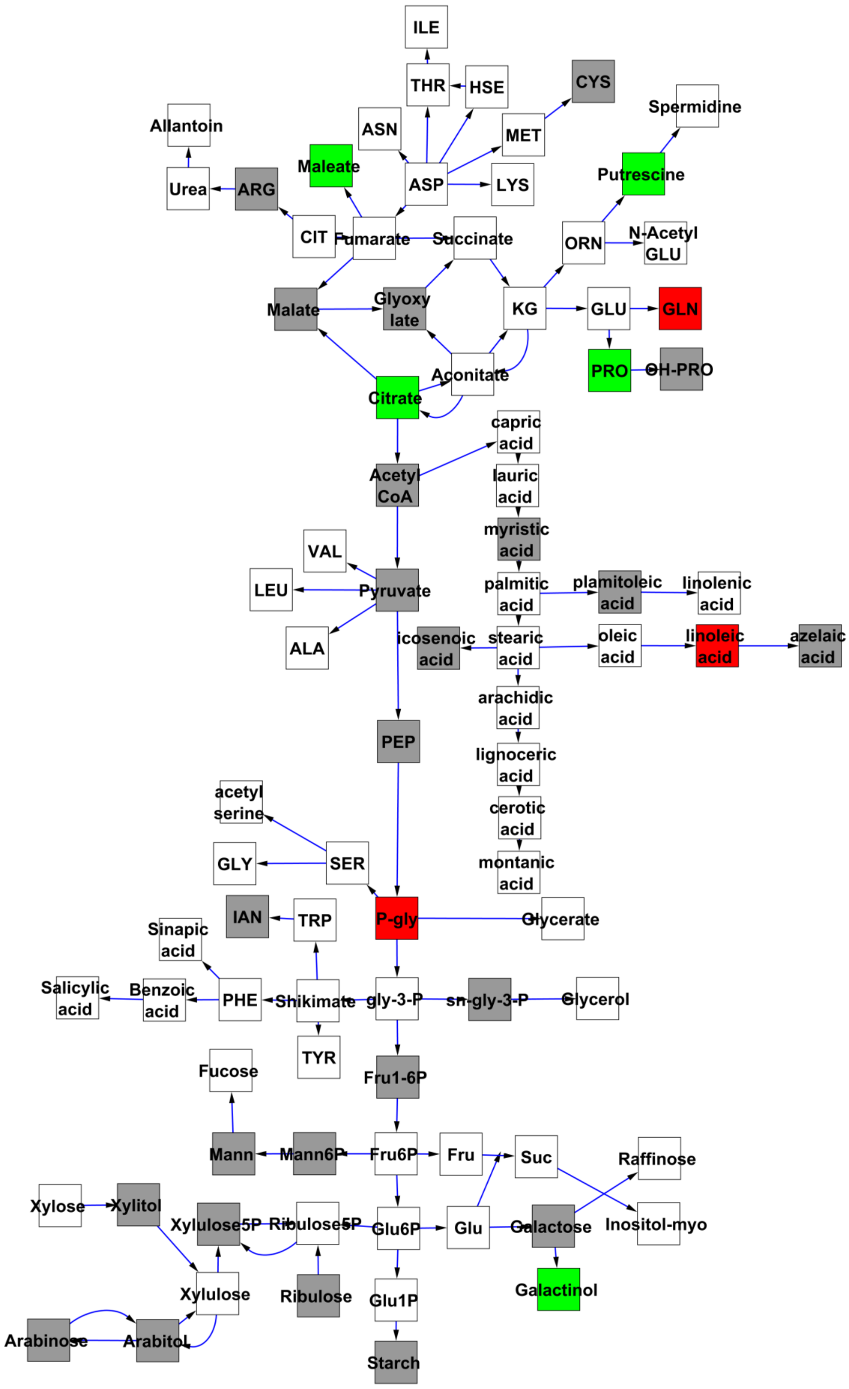

Supplemental Figure S5. Metabolomic Consequence of Variation at Hotspot RG140.

A map of central metabolism was created in Cytoscape and used to plot the estimated allele effect of genetic variation across primary metabolites. A red box shows increased metabolite accumulation when the line contains the Teqing allele while green shows increased metabolite accumulation when the line contains the Lemont allele of a QTL. White boxes are metabolites that were detected but not significantly influenced by the specific QTL and gray boxes show metabolites that were not detected. The QTL locus is named per the nomenclature shown in Figure 4.

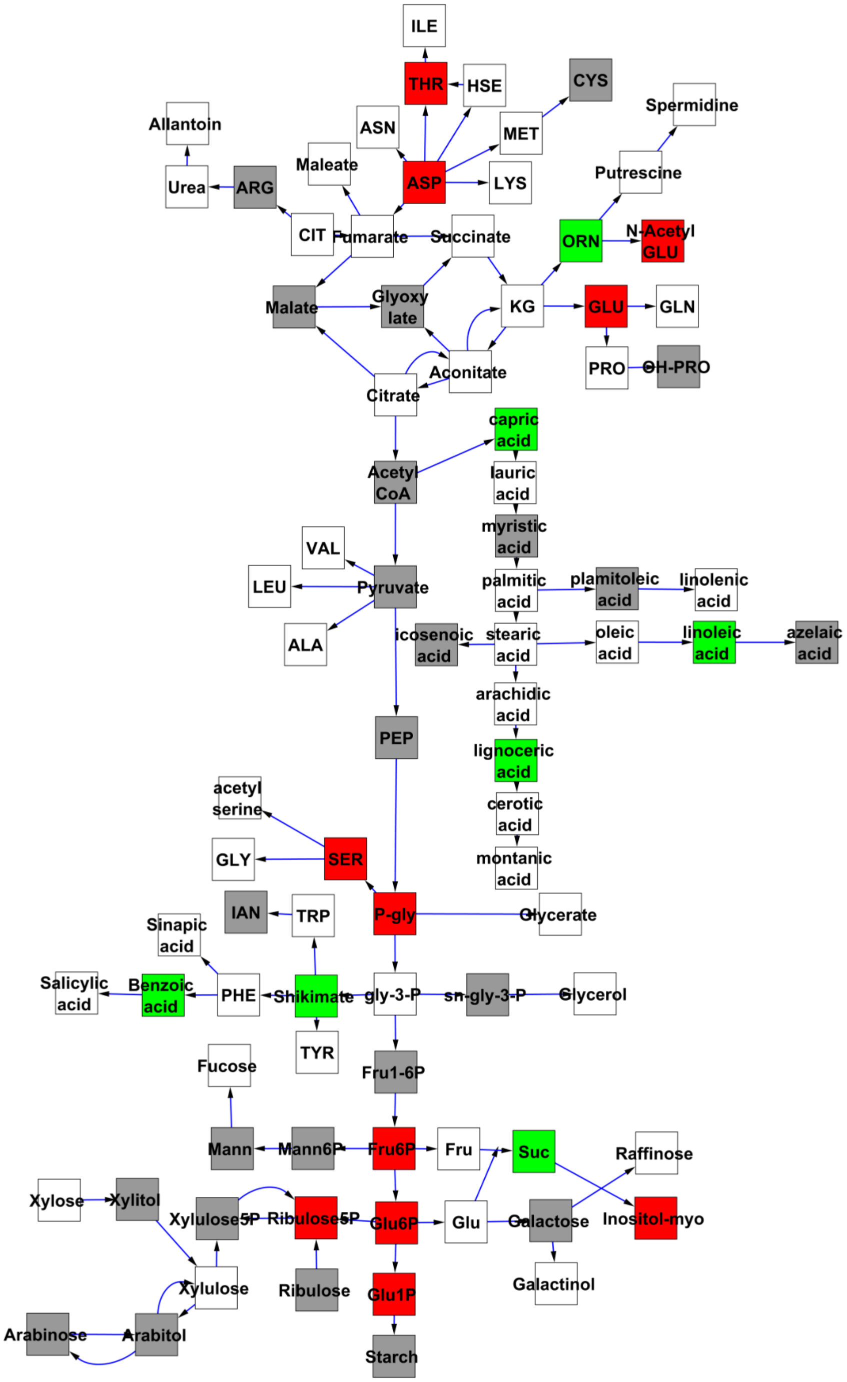

Supplemental Figure S6. Metabolomic Consequence of Variation at Hotspot RZ382.

A map of central metabolism was created in Cytoscape and used to plot the estimated allele effect of genetic variation across primary metabolites. A red box shows increased metabolite accumulation when the line contains the Teqing allele while green shows increased metabolite accumulation when the line contains the Lemont allele of a QTL. White boxes are metabolites that were detected but not significantly influenced by the specific QTL and gray boxes show metabolites that were not detected The QTL locus is named per the nomenclature shown in Figure 4.

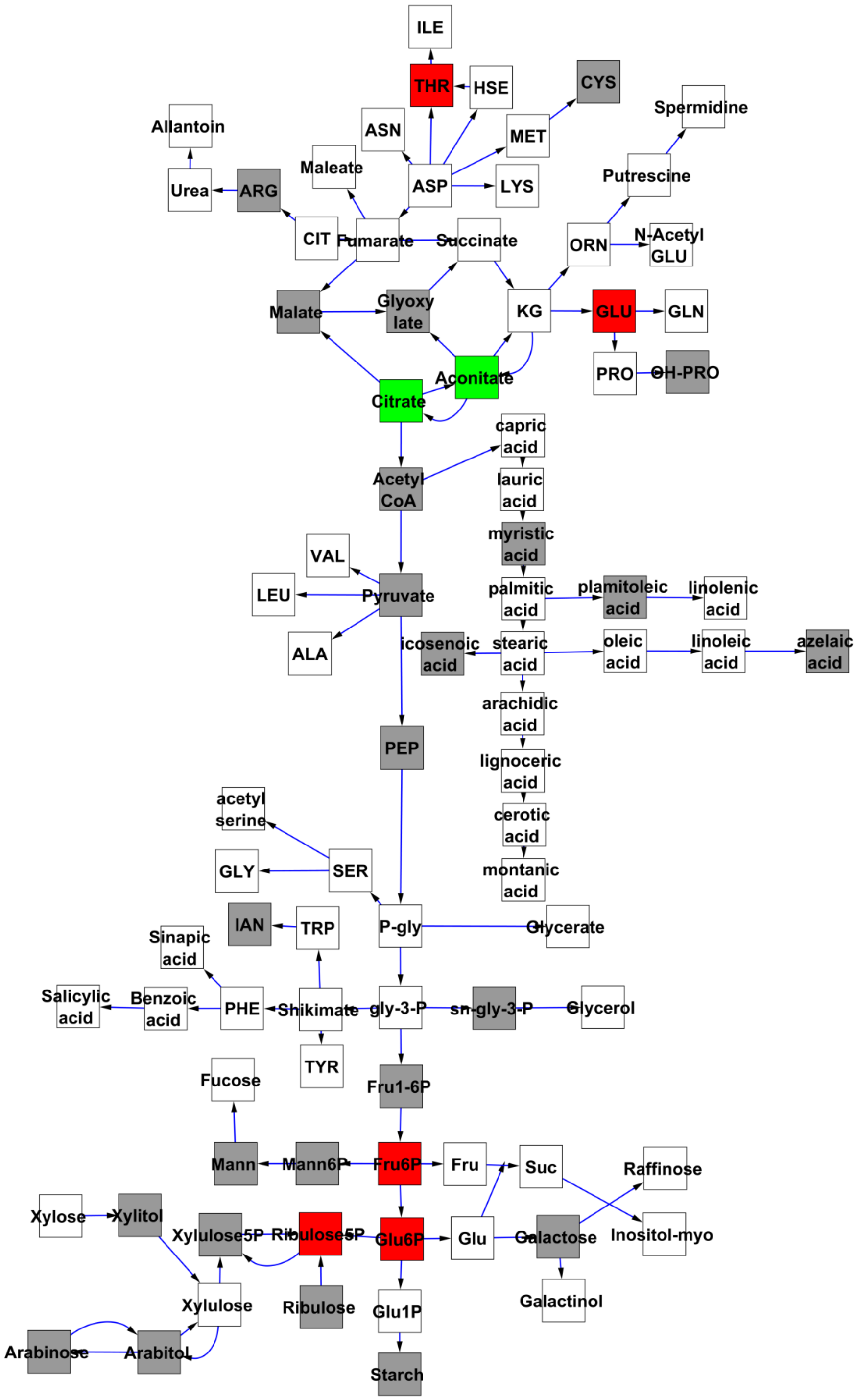

Supplemental Figure S7. Metabolomic Consequence of Variation at Hotspot CDO118.

A map of central metabolism was created in Cytoscape and used to plot the estimated allele effect of genetic variation across primary metabolites. A red box shows increased metabolite accumulation when the line contains the Teqing allele while green shows increased metabolite accumulation when the line contains the Lemont allele of a QTL. White boxes are metabolites that were detected but not significantly influenced by the specific QTL and gray boxes show metabolites that were not detected. The QTL locus is named per the nomenclature shown in Figure 4.

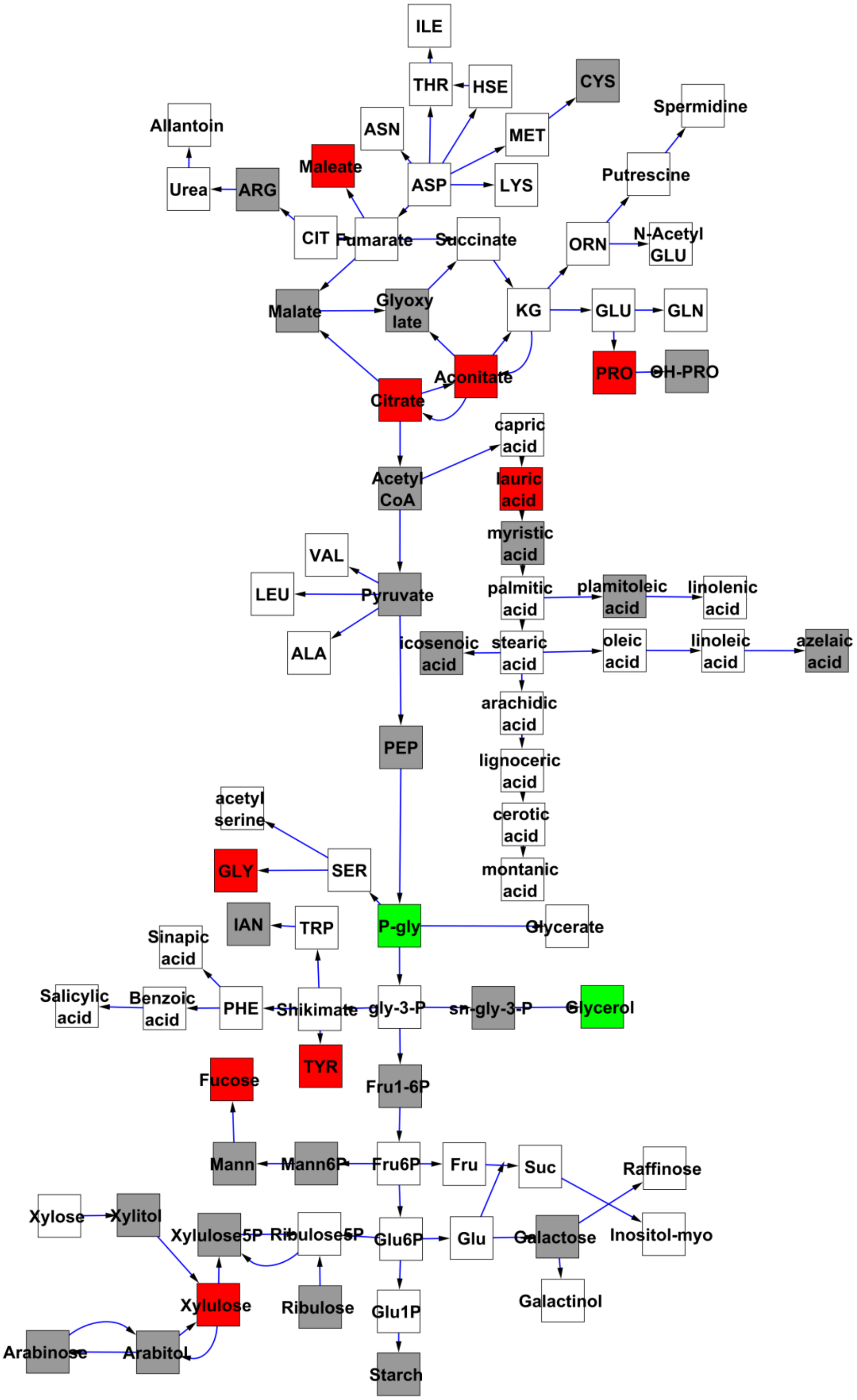

Supplemental Figure S8. Metabolomic Consequence of Variation at Hotspot RG1094e.

A map of central metabolism was created in Cytoscape and used to plot the estimated allele effect of genetic variation across primary metabolites. A red box shows increased metabolite accumulation when the line contains the Teqing allele while green shows increased metabolite accumulation when the line contains the Lemont allele of a QTL. White boxes are metabolites that were detected but not significantly influenced by the specific QTL and gray boxes show metabolites that were not detected. The QTL locus is named per the nomenclature shown in Figure 4.

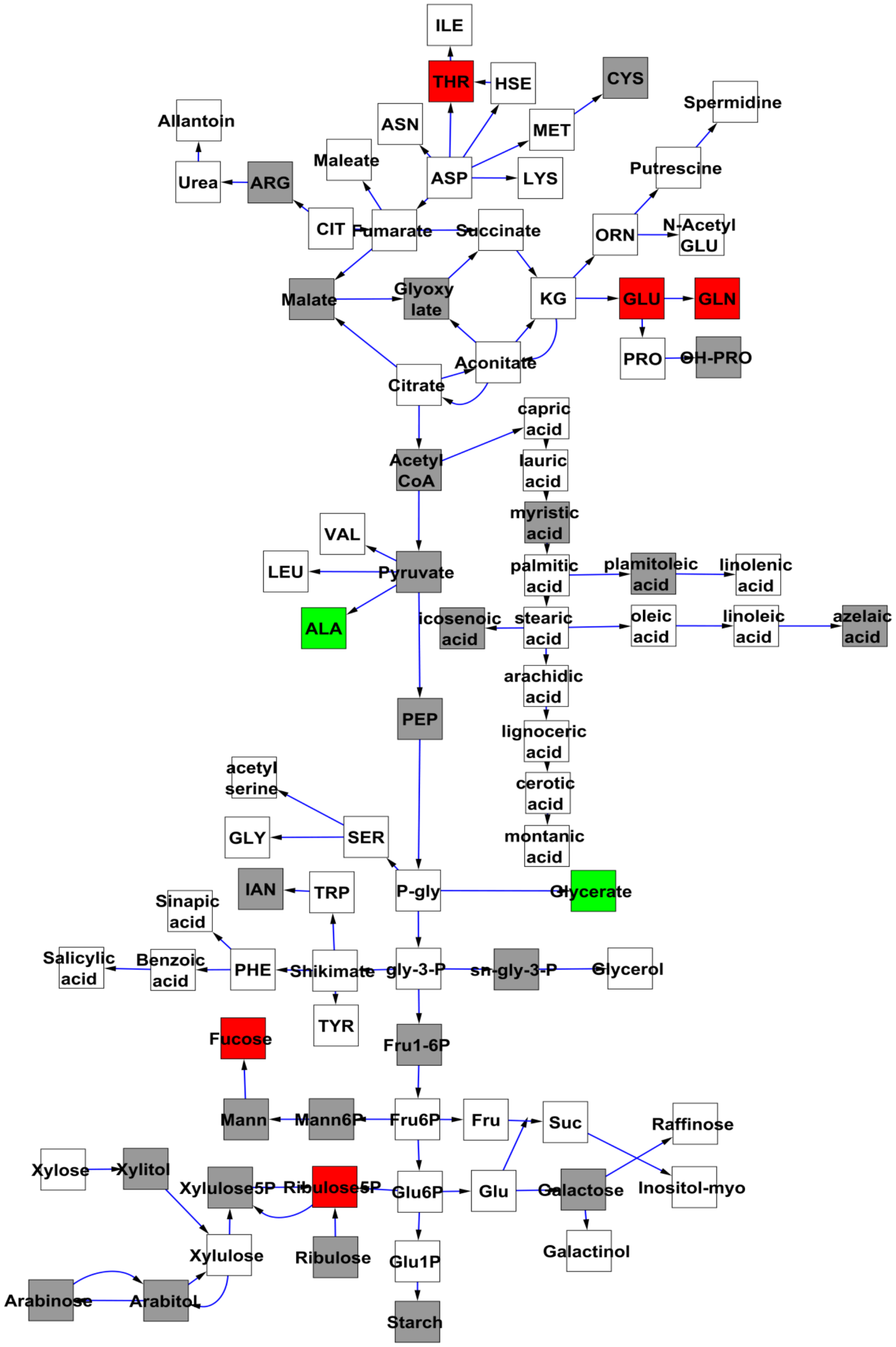

Supplemental Figure S9. Metabolomic Consequence of Variation at Hotspot G294d.

A map of central metabolism was created in Cytoscape and used to plot the estimated allele effect of genetic variation across primary metabolites. A red box shows increased metabolite accumulation when the line contains the Teqing allele while green shows increased metabolite accumulation when the line contains the Lemont allele of a QTL. White boxes are metabolites that were detected but not significantly influenced by the specific QTL and gray boxes show metabolites that were not detected. The QTL locus is named per the nomenclature shown in Figure 4.

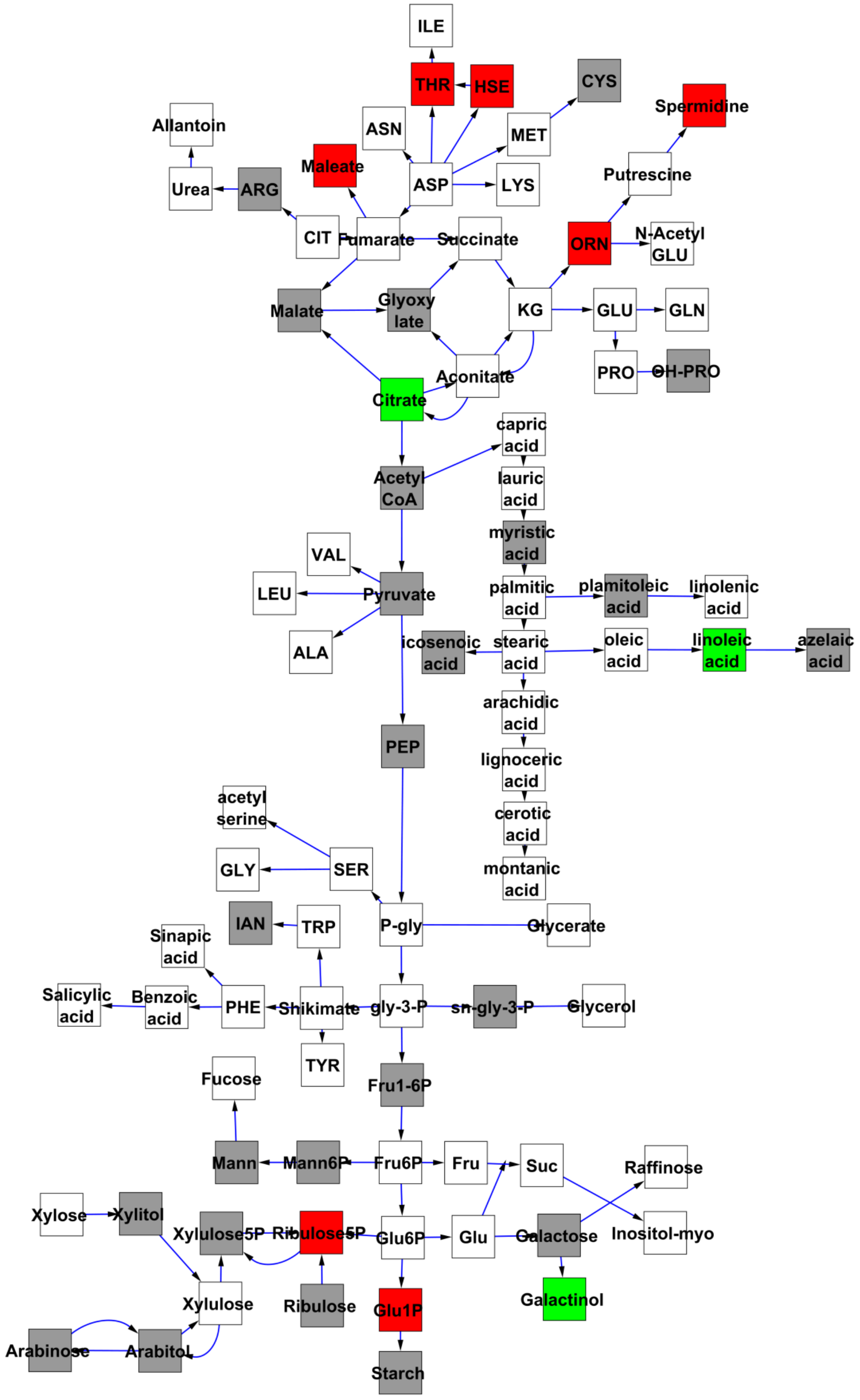

Supplemental Figure S10. Metabolomic Consequence of Variation at Hotspot G20.

A map of central metabolism was created in Cytoscape and used to plot the estimated allele effect of genetic variation across primary metabolites. A red box shows increased metabolite accumulation when the line contains the Teqing allele while green shows increased metabolite accumulation when the line contains the Lemont allele of a QTL. White boxes are metabolites that were detected but not significantly influenced by the specific QTL and gray boxes show metabolites that were not detected. The QTL locus is named per the nomenclature shown in Figure 4.

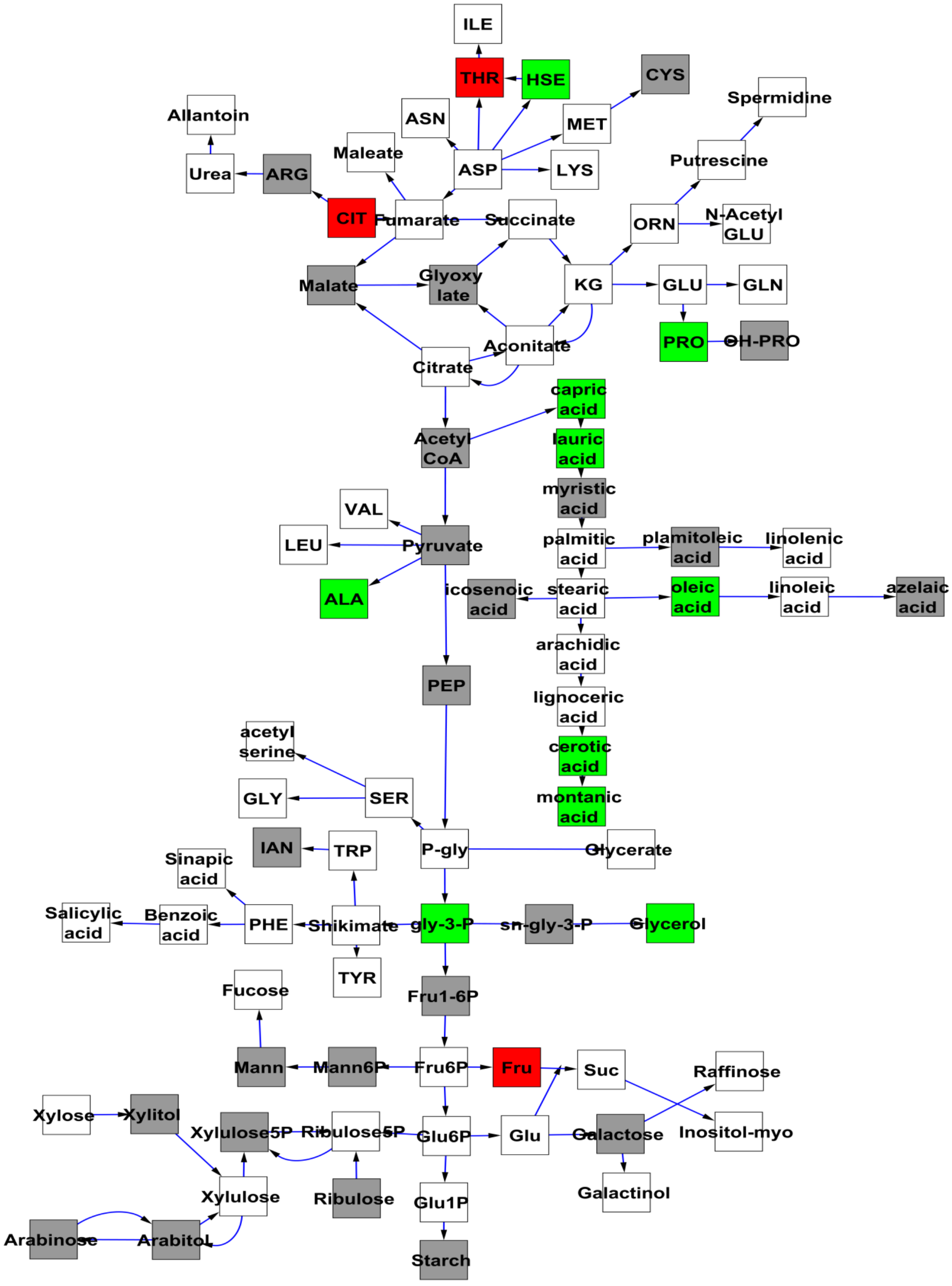

Supplemental Figure S11. Metabolomic Consequence of Variation at Hotspot RZ777.

A map of central metabolism was created in Cytoscape and used to plot the estimated allele effect of genetic variation across primary metabolites. A red box shows increased metabolite accumulation when the line contains the Teqing allele while green shows increased metabolite accumulation when the line contains the Lemont allele of a QTL. White boxes are metabolites that were detected but not significantly influenced by the specific QTL and gray boxes show metabolites that were not detected. The QTL locus is named per the nomenclature shown in Figure 4.

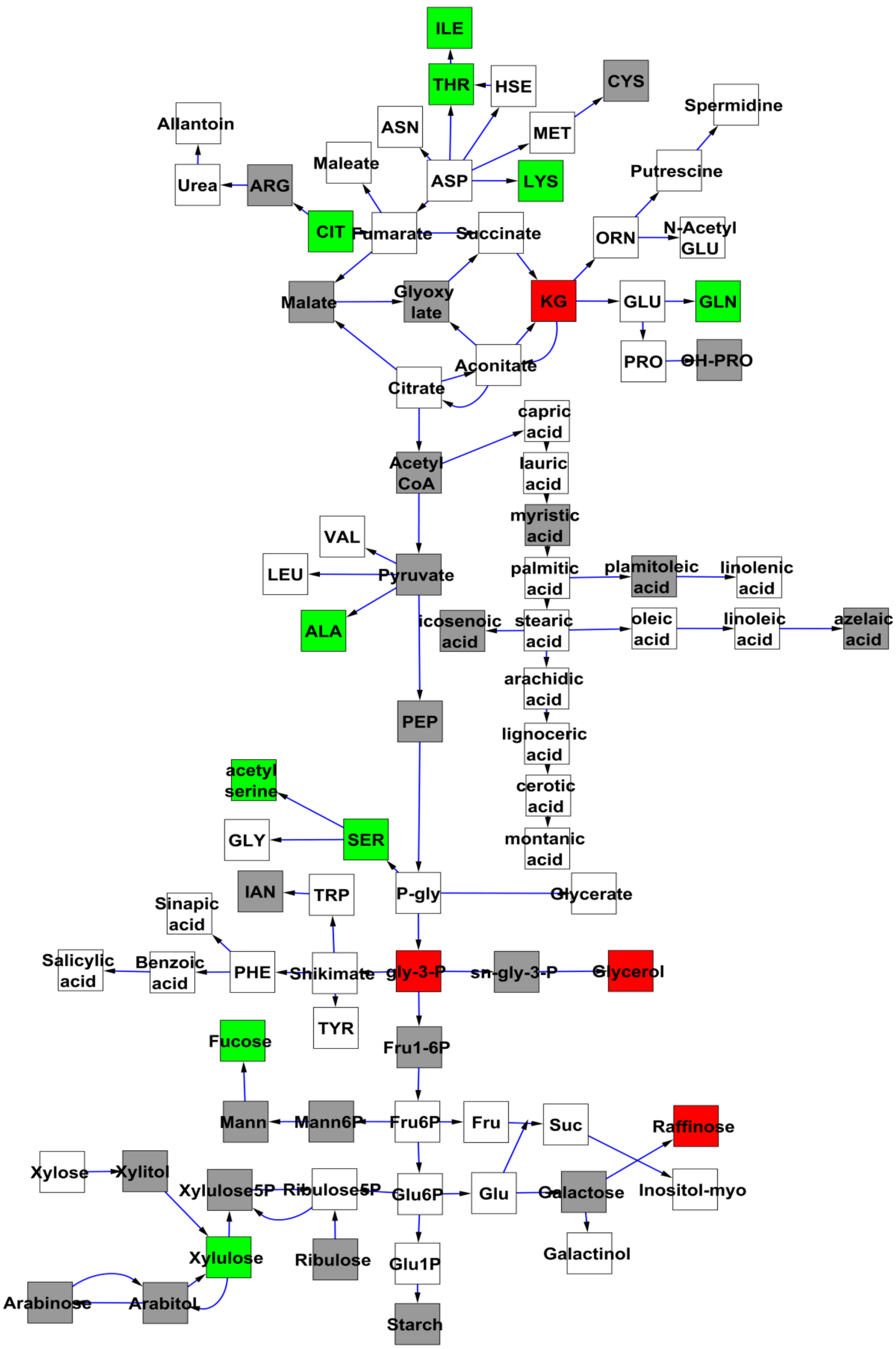

Supplemental Figure S12. Metabolomic Consequence of Variation at Hotspot RZ400.

A map of central metabolism was created in Cytoscape and used to plot the estimated allele effect of genetic variation across primary metabolites. A red box shows increased metabolite accumulation when the line contains the Teqing allele while green shows increased metabolite accumulation when the line contains the Lemont allele of a QTL. White boxes are metabolites that were detected but not significantly influenced by the specific QTL and gray boxes show metabolites that were not detected. The QTL locus is named per the nomenclature shown in Figure 4.

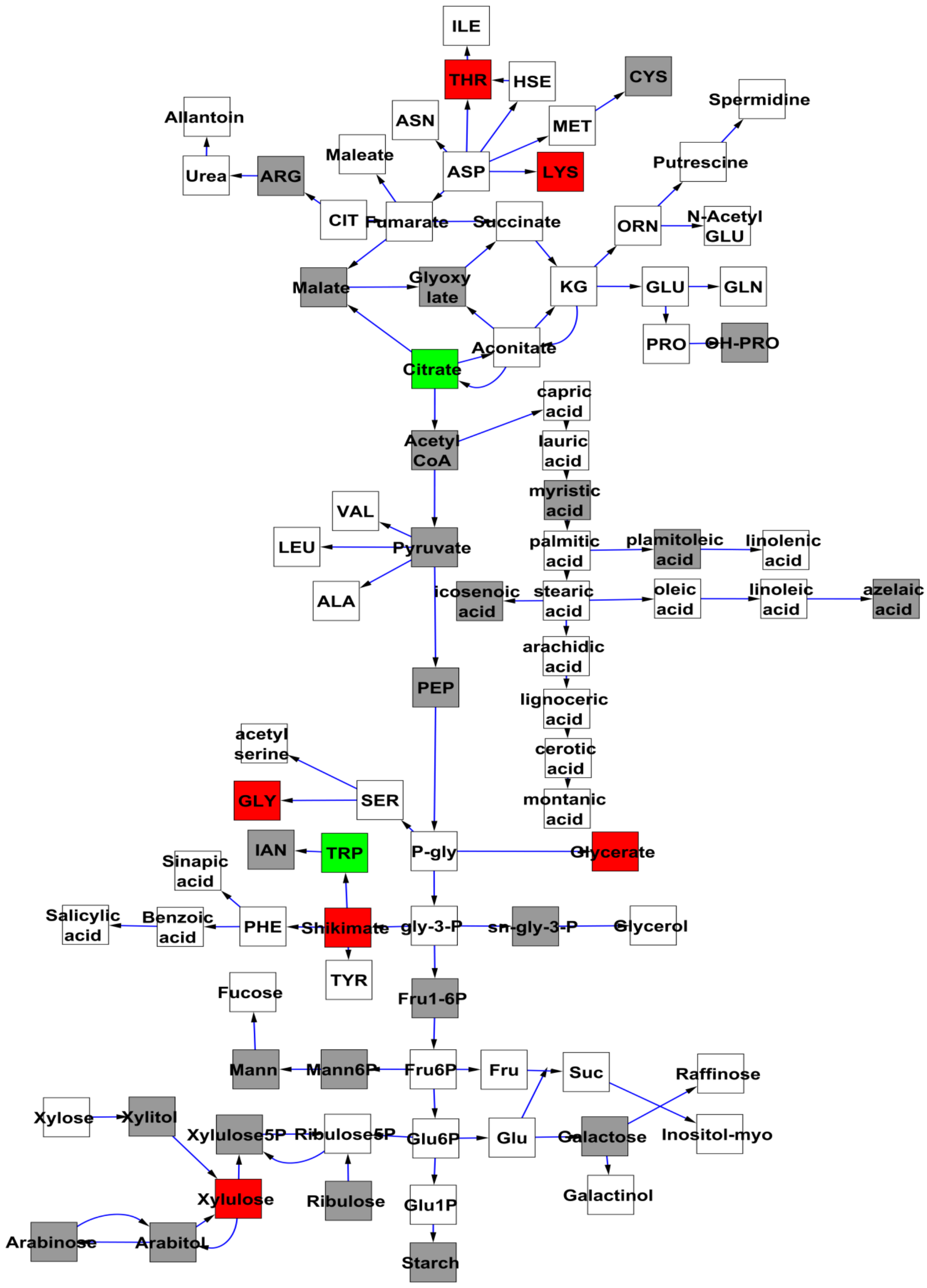

Supplemental Figure S13. Metabolomic Consequence of Variation at Hotspot RZ525a.

A map of central metabolism was created in Cytoscape and used to plot the estimated allele effect of genetic variation across primary metabolites. A red box shows increased metabolite accumulation when the line contains the Teqing allele while green shows increased metabolite accumulation when the line contains the Lemont allele of a QTL. White boxes are metabolites that were detected but not significantly influenced by the specific QTL and gray boxes show metabolites that were not detected. The QTL locus is named per the nomenclature shown in Figure 4.

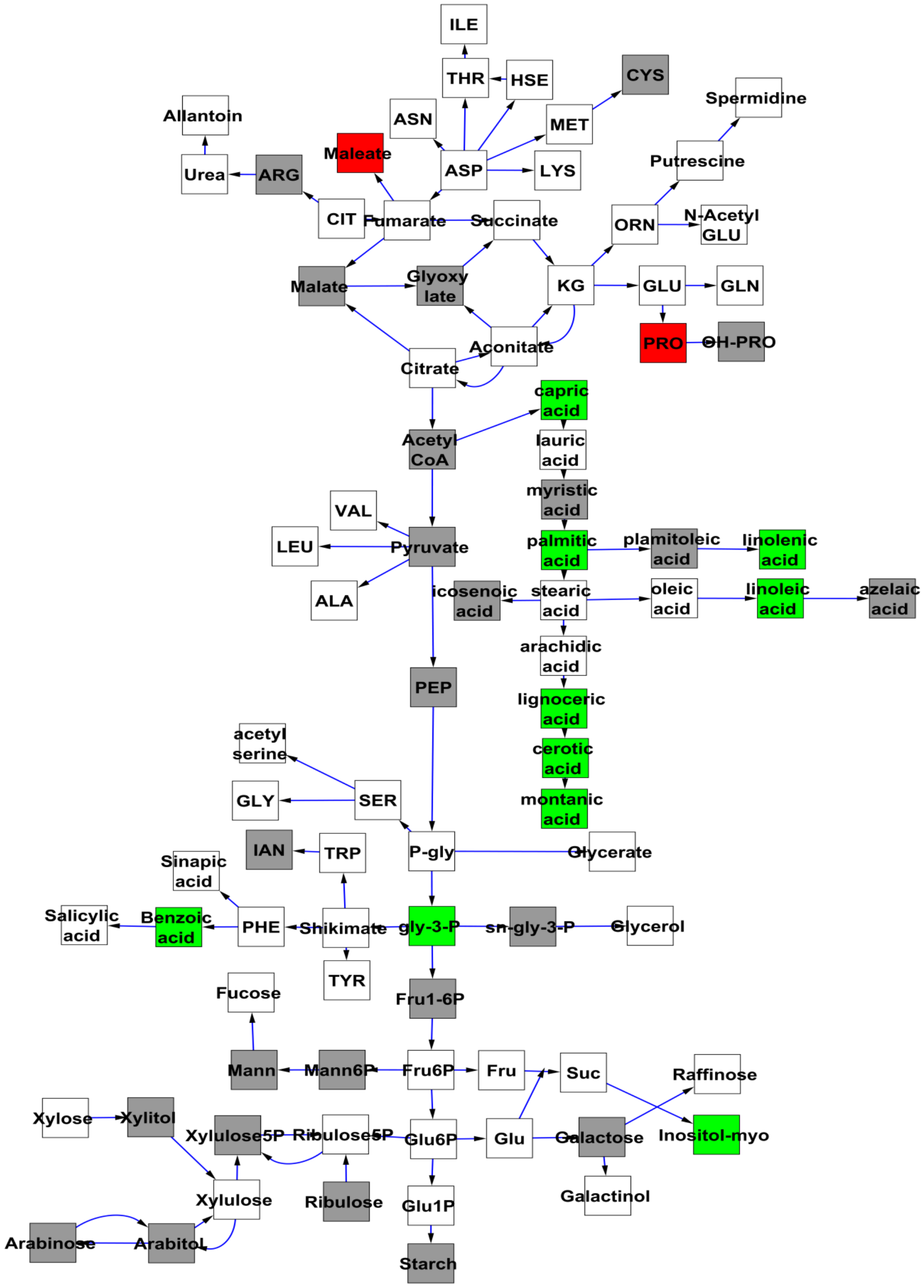

Supplemental Figure S14. Metabolomic Consequence of Variation at Hotspot G193.

A map of central metabolism was created in Cytoscape and used to plot the estimated allele effect of genetic variation across primary metabolites. A red box shows increased metabolite accumulation when the line contains the Teqing allele while green shows increased metabolite accumulation when the line contains the Lemont allele of a QTL. White boxes are metabolites that were detected but not significantly influenced by the specific QTL and gray boxes show metabolites that were not detected. The QTL locus is named per the nomenclature shown in Figure 4.

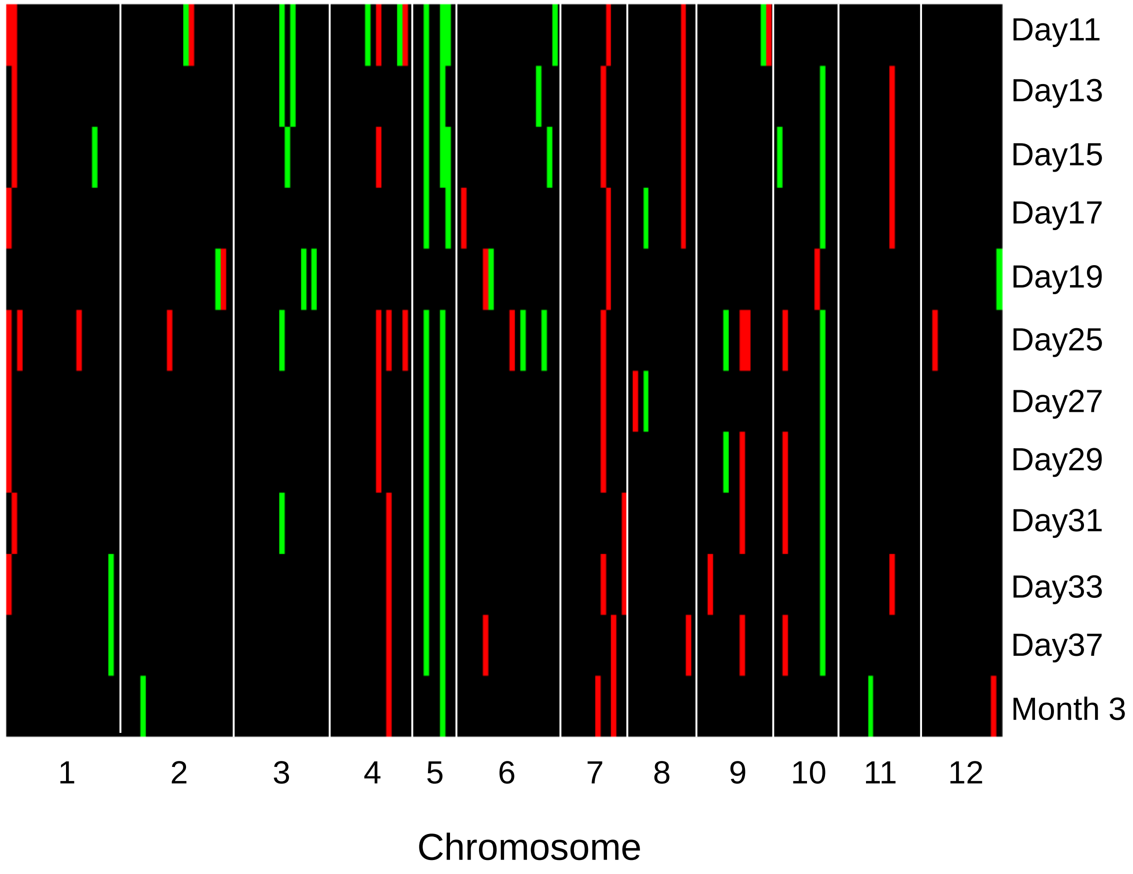

Supplemental Figure S15. Heat Map Showing the Location and Effect of Plant Height QTLs.

The effect of significant QTLs affecting plant height are shown for all height measurements. Red indicates a positive effect of the Teqing allele, while green indicates a positive effect of the Lemont allele at the specific locus. Vertical white lines indicate separation between the chromosomes (1 to 12 from left to right).

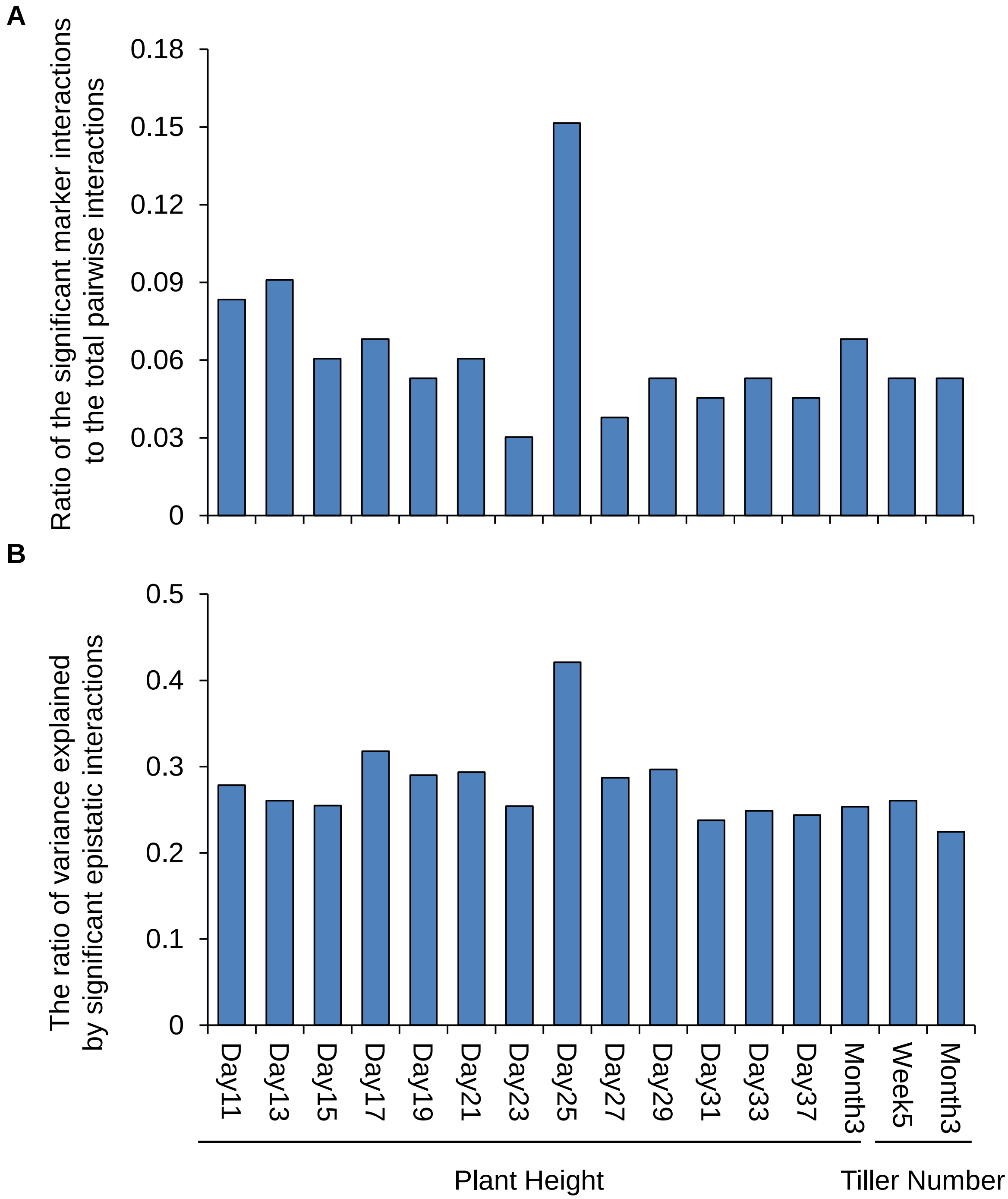

Supplemental Figure S16. Pairwise ANOVA Analysis with Plant Height and Tiller Numbers

Epistatic interactions between QTL hotspots was tested by ANOVA for all plant height and tiller phenotypes. Descriptive summary statistics are presented for all phenotypes.
A: Ratio of the significant marker interactions to the total number of pairwise interactions tested.
B: The ratio of variance explained by significant epistatic interactions in the model with each trait listed at the bottom.
